# Meisoindigo Acts as a Molecular Glue to Target PKMYT1 for Degradation in Chronic Myeloid Leukemia Therapy

**DOI:** 10.1002/advs.202413676

**Published:** 2025-04-25

**Authors:** Zhao‐Xin Zhang, Shu‐Ying Li, Fang‐Fei Li, Qin‐Yan Shi, Cheng‐Yong Tan, Xiao‐Jing Wang, Mi Li, Yun‐Bao Liu, Jing Jin, Yong Li, Shi‐Shan Yu

**Affiliations:** ^1^ State Key Laboratory of Bioactive Substance and Function of Natural Medicines Institute of Materia Medica Chinese Academy of Medical Sciences and Peking Union Medical College Beijing 100050 China

**Keywords:** activity‐based protein profiling, chronic myeloid leukemia, E3 ligase TRIM25, molecular glue, PKMYT1

## Abstract

Meisoindigo (Mei) has been clinically utilized for the treatment of chronic myeloid leukemia (CML), yet the precise molecular targets by which it exerts effects remain unclear. Through activity‐based protein profiling (ABPP), the protein kinase, membrane‐associated tyrosine/threonine 1 (PKMYT1) is identified as a direct target of Mei. Specifically, Mei forms a selective and reversible covalent bond with the Cys301 residue of PKMYT1, triggering its K48‐linked polyubiquitination and accelerating proteasomal degradation, which is mediated by the E3 ligase TRIM25. The study reveals that Mei acts as a molecular glue, enhancing the interaction between PKMYT1 and TRIM25 by approximately 30‐fold, thereby facilitating efficient PKMYT1 degradation. Further investigations reveal the pivotal role of PKMYT1 in cell growth. Knockdown of PKMYT1 in K562 cells induces G2/M phase arrest, enhances early apoptosis, and inhibits cell proliferation. In an orthotopic xenograft model, PKMYT1 knockdown delays leukemia progression and reduces lymph node metastasis, reinforcing its role in CML progression and metastasis. These findings provide a molecular rationale for the clinical efficacy of Mei and highlight PKMYT1 as a promising therapeutic target for CML. Additionally, it offers a valuable scaffold and inspiration for the development of novel molecular glue‐based protein degraders.

## Graphical abstract

Meisoindigo targets PKMYT1 for degradation by acting as a molecular glue that enhances PKMYT1‐TRIM25 interaction, leading to K48‐linked ubiquitination and subsequent proteasomal degradation, thereby exerting therapeutic effects in chronic myeloid leukemia.

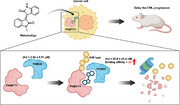

## 1 Introduction

The Wee family of protein kinases plays important regulatory roles in cell cycle progression during mitosis.^[^ ^1^ ^]^ PKMYT1 is a key member of the Wee family,^[^ ^2^ ^]^ and its overexpression is closely related to the occurrence, progression, metastasis and poor prognosis of a variety of solid tumors, including lung cancer,^[^ ^3^ ^]^ gastric cancer,^[^ ^4^ ^]^ liver cancer,^[^ ^5^ ^]^ esophageal cancer,^[^ ^6^ ^]^ breast cancer,^[^ ^7^ ^]^ ovarian cancer,^[^ ^8^ ^]^ colorectal cancer,^[^ ^9^ ^]^ hepatocellular carcinoma,^[^ ^5^ ^]^ and osteosarcoma.^[^ ^10^ ^]^ Consequently, PKMYT1 is a promising therapeutic target for anticancer therapy.^[^ ^5^, ^11^ ^]^ Nevertheless, the development of effective strategies to inhibit PKMYT1 has been hindered by the scarcity of potent and selective inhibitors,^[^ ^11^, ^12^ ^]^ which highlights the urgent need for innovative therapeutic approaches.

Targeted protein degradation (TPD) has emerged as a promising therapeutic strategy for addressing the challenge of “undruggable” proteins.^[^ ^13^ ^]^ This approach harnesses the cellular machinery responsible for protein homeostasis,^[^ ^13a^ ^]^ such as the ubiquitin‐proteasome system (UPS) and the autophagy‐lysosome pathway (ALP),^[^ ^14^ ^]^ to induce the selective degradation of disease‐associated proteins. Among the various TPD modalities, molecular glues and proteolysis‐targeting chimeras (PROTACs) have garnered considerable attention because of their ability to target native, unmodified proteins.^[^ ^13^, ^15^ ^]^ Molecular glues, in particular, have shown potential in stabilizing or inducing protein‐protein interactions (PPIs) between a target protein and an E3 ubiquitin ligase, thereby facilitating the ubiquitination and subsequent degradation of the target protein.^[^ ^13^, ^16^ ^]^ This strategy has been applied in the development of therapies for various cancers, including leukemia,^[^ ^17^ ^]^ liver cancer^[^ ^18^ ^]^ and colorectal cancer.^[^ ^19^ ^]^

Meisoindigo (Mei), a derivative of the natural product indirubin, has been used in the clinical management of chronic myeloid leukemia (CML) in China since 1992.^[^ ^20^ ^]^ Compared with its predecessor, indirubin, it has superior efficacy and fewer side effects.^[^ ^20c^ ^]^ Despite its established clinical utility, the precise molecular target and mechanism of action of Mei in CML treatment remain unclear. In this study, we revealed a novel role for Mei as a molecular glue that induces the degradation of PKMYT1 by promoting its interaction with the E3 ubiquitin ligase TRIM25. Our findings not only elucidate the mechanism by which Mei exerts its antitumor effects but also provide a blueprint for the development of molecular glue‐based therapeutics targeting PKMYT1, offering new avenues for the treatment of CML and potentially other cancers.

## 2 Results

### 2.1 Identification of Potential Targets for Meisoindigo through Activity‐Based Proteomics

**Figure 1:**
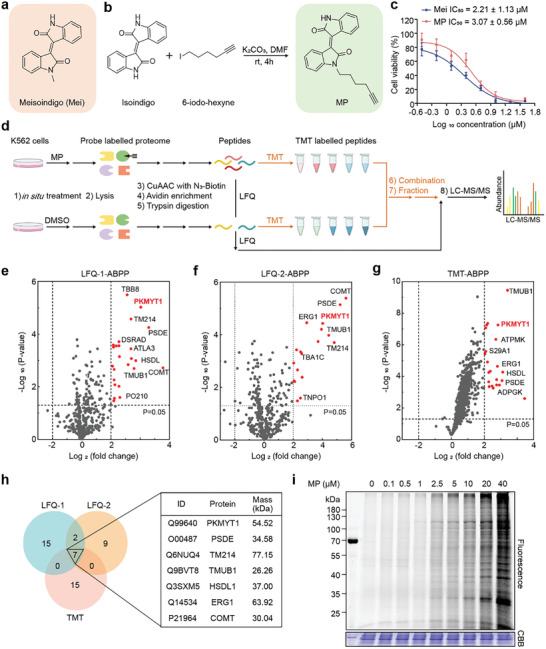
Activity‐based proteomic identification of potential targets for meisoindigo. a) Chemical structure of meisoindigo (Mei). b) Synthesis of a Mei‐alkyne probe (MP). c) Cell viability of K562 cells treated with Mei or MP for 72 h (n = 3). d) Workflow for label‐free (LFQ) and tandem mass tag (TMT) quantitative proteomics to identify MP‐labeled proteins in K562 cells. e,f) Volcano plots of the first and second label‐free quantitative proteomics with MP (5 µM)/DMSO (negative control) (n = 3). g) Volcano plot of TMT quantitative proteomics with MP (5 µM)/DMSO (negative control) (n = 5). h) Venn diagram summarizing overlapping proteins from the quantitative proteomics data. i) Concentration‐dependent in situ fluorescence labeling of MP in K562 cells. The data are presented as the means ± SDs. Statistical significance was assessed via two‐tailed unpaired Student's t‐test. Figure (d) created with BioRender.com.

The specific molecular targets of Mei (**Figure** **1**a), which have been utilized clinically in China since 1992 for the treatment of chronic myeloid leukemia (CML), remain unclear.^[^ ^20d^ ^]^ To identify its potential targets and gain insights into the molecular mechanisms underlying its therapeutic effects, an active Mei‐based probe (MP) bearing an alkyne moiety was designed and synthesized (Figure 1b; Scheme S1, Supporting Information). The results of the cytotoxic activity assays revealed that MP activity was comparable to Mei activity in K562 cells (Figure 1c) and other hematologic cell lines (Figure S1a,b, Supporting Information), indicating that the addition of the alkyne group did not significantly alter the efficacy of Mei. Confocal microscopy confirmed that MP effectively labeled K562 cells, and the labeling intensity was reduced upon pretreatment with Mei (Figure S1c, Supporting Information), revealing a shared target‐binding property between Mei and the probe MP.

Using activity‐based protein profiling (ABPP), a chemical proteomics approach,^[^ ^21^ ^]^ we aimed to identify the direct targets of Mei. In activity‐based protein profiling (ABPP), TAMRA (in fluorescence labeling experiments) and biotin (in quantitative proteomics experiments) serve as reporting groups. Fluorescence labeling experiments can be used to evaluate the labeling ability of MP and to establish optimal labeling conditions for subsequent quantitative proteomics. In the fluorescence labeling experiments, live K562 cells were treated with MP for 1 h, followed by lysis and click reaction with TAMRA‐azide. Upon SDS‐PAGE and in‐gel fluorescence scanning of the labeled proteomes, as the concentration of MP increased, the labeling intensity gradually intensified (Figure 1i), and MP has good labeling efficiency for intracellular target proteins. In dose‐response fluorescence labeling experiments, a labeling effect at 5 µM was found to be quite sufficient (Figure 1i). Furthermore, according to literature, incubating low concentrations of the probe with high concentrations of proteome increases the likelihood of successfully capturing the primary protein targets.^[^ ^22^ ^]^ Additionally, in time‐course fluorescence labeling experiments, an incubation period of 1 h was sufficient for labeling (Figure S1d, Supporting Information). Therefore, based on these findings, a concentration of 5 µM and an incubation period of 1 h were selected as the optimal experimental conditions for subsequent experiments. Preincubation with Mei effectively blocked MP labeling (Figure S1e, Supporting Information), suggesting that MP can be utilized in target identification experiments (ABPP) for Mei. Specifically, there are two bands in the molecular weight ranges of 100–130 and 55–70 kDa that can be specifically competed by Mei, which can provide insights into the screening of target proteins. Under these conditions, K562 cells treated with MP were lysed, and the MP‐labeled proteins were captured using biotin‐azide via click chemistry. The captured proteins were then enriched, digested into peptides, and analyzed by LC‐MS/MS using either label‐free (LFQ) or tandem mass tag (TMT) quantitative proteomics (Figure 1d).

Comparative analysis of the MP‐captured versus control‐captured proteomes revealed seven proteins that consistently met our filtering criteria (fold change > 4 and p‐value < 0.05) across three independent experiments (Figure 1e–g,h). Notably, none of these seven proteins were in the 100–130 kDa range and only PKMYT1 and ERG1 had molecular weights in the 55–70 kDa range (Figure 1h). Their findings indicate that MP serves as a reliable activity‐based probe for identifying the cellular targets of Mei via ABPP.

### 2.2 PKMYT1 Serves as a Direct Target of Mei

**Figure 2:**
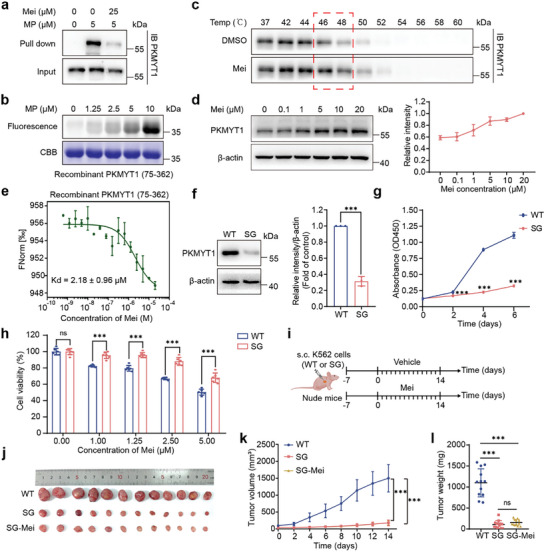
Mei exerts antitumor effects by directly targeting PKMYT1. a) Western blot analysis of the PKMYT1 protein in protein affinity pull‐down assay in K562 cells, and the cells were treated with MP (5 µM) with or without Mei (25 µM). b) Concentration‐dependent fluorescence labeling of MP on recombinant PKMYT1 (75‐362). c) Mei treatment (10 µM) increased the thermal stability of PKMYT1 at the whole‐cell level, as measured by a temperature‐dependent cellular thermal shift assay (CETSA) (n = 3). d) Mei treatment increased the thermal stability of PKMYT1 in cell lysates, as measured by a concentration‐dependent CETSA at 47 °C (n = 3). e) MST assay of Mei binding to recombinant PKMYT1 (75‐362) (n = 3). f) Immunoblotting confirmed PKMYT1 knockdown in K562 cells via the CRISPR/Cas9 system (n = 3). g) Growth curves of wild‐type and PKMYT1‐knockdown K562 cells (n = 3). h) Viability of wild‐type and PKMYT1‐knockdown K562 cells after 48 h Mei treatment at various concentrations (n = 6). i) Schematic diagram of Mei administration in mice bearing wild‐type or PKMYT1‐knockdown K562 tumors. j) Images of tumors from mice (wild‐type or PKMYT1‐knockdown) treated with vehicle or Mei (150 mg kg^−1^) (n = 12). k) Dynamic changes in tumor volume (n = 12). l) Tumor weight (n = 12). The data are presented as the means ± SDs. Statistical significance was assessed via two‐tailed unpaired Student's t‐test. NS, not significant; ***P < 0.001 versus the wild‐type group. Figure (i) created with BioRender.com.

To identify the primary targets among the candidates, we conducted in situ pull‐down assays. Mei exhibited the strongest competitive binding to PKMYT1, achieving near‐complete displacement of the probe label at twice the concentration of the MP (**Figure** **2**a; Figure S2a, Supporting Information). In contrast, the other candidates showed significantly less competitive binding (Figure S2b–g, Supporting Information). These findings provide preliminary validation of PKMYT1 as a potential binding partner of Mei.

Further validation of the interaction between Mei and PKMYT1 was achieved through a series of experiments. Concentration‐dependent fluorescence labeling of recombinant PKMYT1 (75‐362) by MP (Figure 2b), along with robust labeling of full‐length PKMYT1, was observed (Figure S2h, Supporting Information), suggesting the specific binding selectivity of Mei to PKMYT1. Then, we determined the target engagement of Mei in K562 cells by using a cellular thermal shift assay (CETSA). Treatment with Mei markedly increased the thermal stability of PKMYT1 in both cells and lysates in a dose‐dependent manner (Figure 2c,d), suggesting that there was an intracellular interaction between Mei and PKMYT1. Furthermore, microscale thermophoresis (MST) analysis confirmed the direct binding of Mei to recombinant PKMYT1 (75‐362), with a dissociation constant (*Kd*) of 2.18 ± 0.96 µM (Figure 2e). These data provide strong evidence of a specific and direct interaction between Mei and PKMYT1.

To investigate whether the antitumor effects of Mei are mediated by PKMYT1, we used CRISPR‐Cas9 gene editing to knockdown PKMYT1 expression in K562 cells. The knockdown effect was validated via immunoblotting (Figure 2f). PKMYT1 knockdown significantly suppressed K562 cell growth and proliferation (Figure 2g), suggesting its involvement in leukemia progression. Moreover, we observed that Mei‐dependent inhibition of cell proliferation was significantly attenuated in PKMYT1‐knockdown K562 cells (Figure 2h; Figure S2i, Supporting Information). In vivo experiments using an ectopic xenograft model (Figure 2i) revealed that PKMYT1 knockdown substantially inhibited tumor growth, in terms of both volume and weight (Figure 2j–l), compared with that in the group engrafted with wild‐type K562 cells. Notably, Mei treatment (150 mg kg^−1^ for 14 days) did not further suppress the growth of PKMYT1‐knockdown ectopic xenograft tumors compared with that of wild‐type tumors, reinforcing the critical role of PKMYT1 in the antitumor effect of Mei. Collectively, these findings confirm that PKMYT1 is a direct target of Mei that mediates its anticancer effects.

### 2.3 Reversible Covalent Modification of PKMYT1 by Mei

**Figure 3:**
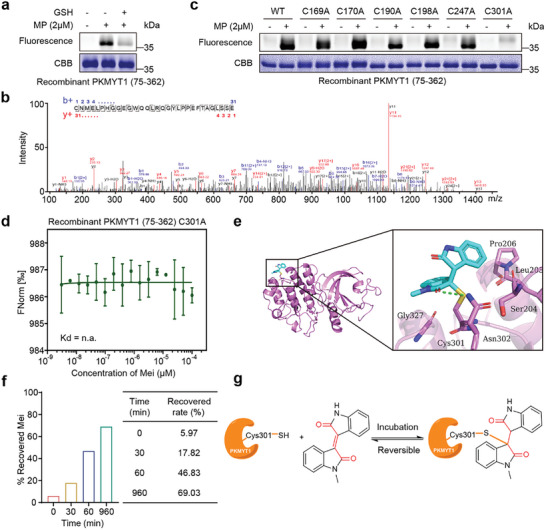
Mei covalently and reversibly binds to the Cys301 residue of PKMYT1. a) Fluorescence labeling of the recombinant PKMYT1 (75‐362) protein revealed the impact of glutathione (GSH) preincubation on MP binding, indicating covalent binding. b) LC‐MS/MS analysis confirms Mei‐mediated modification at the Cys301 residue of PKMYT1 (75‐362). c) Fluorescence labeling of wild‐type and cysteine‐mutated recombinant PKMYT1 (75‐362) proteins by MP. d) MST assay of Mei binding to the recombinant PKMYT1 (75‐362) C301A mutant (n = 3). e) 3D binding mode between PKMYT1 and Mei determined by molecular dynamics (MD) simulation. f) Mei dissociates from the recombinant PKMYT1 (75‐362) protein upon unfolding. g) Proposed mechanism of the interaction between Mei and PKMYT1. For d, the data are presented as the means ± SDs. For e, Mei is colored in cyan, and PKMYT1 is colored violet; the surrounding residues in the binding pocket of PKMYT1 are depicted as magenta sticks; and the hydrogen bonds are depicted as green dashed lines.

The *α*,*β*‐unsaturated ketone moiety, which serves as a Michael acceptor, is vital for the biological activity of numerous compounds because of its ability to form covalent bonds with cysteine thiols in proteins.^[^ ^23^ ^]^ We initially investigated the reactivity potential of the *α*,*β*‐unsaturated ketone structure in Mei. As shown in **Figure** **3**a, preincubation with glutathione (GSH), which contains a thiol group, disrupted the binding between MP and PKMYT1. Moreover, the direct detection of a Michael addition complex formed between Mei and GSH via LC‐MS (Figure S3a, Supporting Information) confirmed that the covalent interaction of Mei with cysteine thiols in PKMYT1 is mediated by its *α*,*β*‐unsaturated ketone moiety. To verify the important role of *α,β*‐unsaturated ketone moiety for the pharmacological activity exerted by Mei. We obtained the compound MR (Figure S6a, Supporting Information), which is a reduction product of Mei. It was found that MR had no cytotoxic activity in the range of 40 µM and had no effect on PKMYT1 protein levels in K562 cells, suggesting that *α*,*β*‐unsaturated ketone moiety is essential for Mei to exert cytotoxic activity (Figure S6b, Supporting Information) as well as to promote PKMYT1 degradation (Figure S6c,d, Supporting Information).

We subsequently delved into the direct binding site of Mei within PKMYT1. LC‐MS/MS analysis of Glu‐C‐digested peptides from the recombinant PKMYT1 (75‐362) protein treated with Mei revealed the labeling of Cys301 (Figure 3b). We scanned the PKMYT1 (75‐362) protein sequence and identified six cysteine residues (Figure S4, Supporting Information). To validate that Cys301 is the binding site for Mei in PKMYT1, we individually mutated all six cysteines in recombinant PKMYT1 (75‐362) to alanine. Fluorescence labeling of the mutant proteins with MP revealed that only the Cys301 mutation abolished the binding of MP to PKMYT1 (Figure 3c). Furthermore, MST experiments confirmed that Mei no longer interacts with the Cys301A‐mutated recombinant PKMYT1 (75‐362) protein (Figure 3d). To elucidate the intricate structural dynamics of the Mei‐PKMYT1 complex, we performed a computational analysis involving 200 ns molecular dynamics simulations. In the final stable conformation, Mei adopts an “upright” conformation binding to Cys301, with its two indole rings extending outward from the protein surface. This particular orientation appears to be conducive to facilitating interactions with other proteins. Moreover, the hydrogen bond formed between an amide carbonyl group of Mei and Asn302 plays a critical role in stabilizing the conformation of Mei‐PKMYT1 (Figure 3e). Collectively, these findings confirm the selective covalent binding of Mei to the Cys301 residue of PKMYT1.

Additionally, we observed that during the incubation of Mei with cysteine, the color of the reaction system gradually faded from reddish‐brown, indicating the disruption of Mei's conjugated system. After the reaction, the mixture was extracted with an ethyl acetate:water (1:1) solvent system. Interestingly, the color of the organic phase gradually changed from light red to dark red over time (Figure S3b, Supporting Information), suggesting the gradual restoration of Mei's conjugated system, which indicates that the reaction between Mei and cysteine is reversible.

To investigate the reversibility of the reaction between Mei and thiols, we reacted Mei with *β*‐mercaptoethanol (BME) and obtained adducts of Mei‐BME (Figure S3c, Scheme S2, Supporting Information). Notably, this adduct undergoes decomposition, and we monitored its *β*‐elimination rate in PBS at pH 7.4 via LC‐MS/MS (Figure S3d, Supporting Information). The half‐life of the Mei‐BME adduct was determined to be 112.9 min (K_1/2_ = 0.006139 min^−1^). Additionally, we assessed the dissociation rate of Mei from recombinant PKMYT1 (75‐362) during protein denaturation and recovered 69.03% of Mei after 16 h (Figure 3f). These results indicate that Mei forms a reversible covalent bond with the Cys301 residue of PKMYT1 (Figure 3g).

### 2.4 PKMYT1 Undergoes Degradation via K48‐Linked Ubiquitination

**Figure 4:**
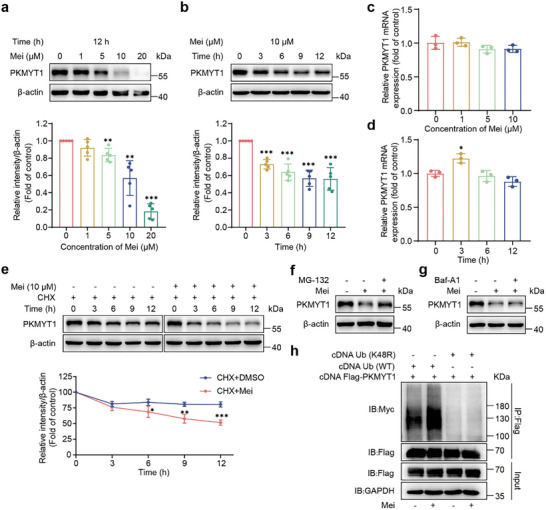
Mei promotes PKMYT1 degradation via K48‐linked ubiquitination. a) Western blot analysis of PKMYT1 protein levels in K562 cells treated with different concentrations of Mei for 12 h (n = 5). b) Western blot analysis of PKMYT1 protein levels in K562 cells treated with Mei (10 µM) at different time points (n = 5). c) Real‐time qPCR assessment of PKMYT1 mRNA levels in K562 cells treated with different concentrations of Mei for 4 h (n = 3). d) Time‐dependent qPCR analysis of PKMYT1 mRNA levels in K562 cells following treatment with Mei (10 µM) (n = 3). e) Western blot analysis of PKMYT1 degradation in K562 cells treated with cycloheximide (CHX) with or without Mei (10 µM) at different time points (n = 3). f) A proteasome inhibitor rescued the reduction of PKMYT1 in K562 cells, and the cells were treated with Mei alone or in combination with MG‐132 (10 µM) for 6 h. g) Effect of the lysosome inhibitor bafilomycin A1 (Baf‐A1, 200 nM) on Mei‐mediated PKMYT1 degradation in K562 cells. h) Co‐IP assay demonstrating Mei‐induced K48‐linked ubiquitination of PKMYT1. The data are presented as the means ± SDs. Statistical significance was assessed via two‐tailed unpaired Student's t‐test, *P < 0.05, **P < 0.01, ***P < 0.001 versus the control group.

PKMYT1, a member of the Wee family of serine/threonine kinases, phosphorylates threonine 14 (Thr14) of cyclin‐dependent kinase 1 (CDK1), thereby inhibiting its ability (when complexed with cyclin B) to trigger mitosis.^[^ ^2^ ^]^ Given that Mei binds directly to PKMYT1, we conducted an enzyme activity assay to determine whether Mei inhibits the enzymatic activity of PKMYT1. Since Mei binds PKMYT1 outside its catalytic domain, it was unsurprising that neither Mei nor MP inhibits PKMYT1's enzymatic activity, contrasting with the reference inhibitor PD0166285,^[^ ^24^ ^]^ which had a half‐maximal inhibitory concentration (IC_50_) of 16.42 nM in our test (Figure S5a, Supporting Information). However, compared with DMSO‐treated control cells, K562 cells exposed to various concentrations of Mei presented markedly lower PKMYT1 protein levels (**Figure** **4**a,b), accompanied by decreased phosphorylation of CDK1 at Thr14 (Figure S5b, Supporting Information). Importantly, Mei treatment did not alter PKMYT1 mRNA expression (Figure 4c,d), suggesting that a post‐transcriptional regulatory mechanism underlies PKMYT1 downregulation. These results suggest that Mei serves as a small‐molecule PKMYT1 degrader. To assess the reversibility of PKMYT1 degradation at the cellular level, a washout experiment was conducted. After K562 cells were treated with Mei (10 µM) for 12 h, they were washed twice with PBS and incubated for an additional 12 h. Western blot analysis revealed complete restoration of PKMYT1 expression to baseline levels (Figure S5c, Supporting Information), indicating the reversibility of Mei‐induced PKMYT1 degradation in K562 cells. This observation aligns with the reversible covalent interaction of Mei with the Cys301 residue of PKMYT1.

Cycloheximide (CHX), a protein synthesis inhibitor, was used to further confirm that Mei promotes PKMYT1 degradation. When treatment with CHX alone, PKMYT1 levels remained stable for 3–12 h; however, cotreatment with Mei and CHX increased the PKMYT1 degradation rate, further supporting that the reduction in PKMYT1 protein levels was due to an increase in degradation (Figure 4e). Regulated protein degradation primarily occurs via the UPS and ALP, with the UPS responsible for eliminating more than 80% of the protein in eukaryotic cells.^[^ ^13^, ^14^ ^]^ We found that the proteasome inhibitor MG‐132 significantly blocked the Mei‐induced decrease in PKMYT1 protein levels (Figure 4f), whereas the lysosomal inhibitor bafilomycin A1 (Baf‐A1) had no such effect (Figure 4g), which indicates that PKMYT1 degradation does not occur via the ALP. Additionally, experiments conducted at various time points (Figure S5d, Supporting Information) confirmed that MG‐132 but not Baf‐A1 prevented the time‐dependent degradation of PKMYT1 in K562 cells treated with CHX. Collectively, these data indicate that PKMYT1 is primarily degraded via the UPS pathway.

Protein ubiquitination, a post‐translational modification of lysine residues, is closely associated with protein degradation and plays an important role in many cellular processes.^[^ ^25^ ^]^ There are currently seven known residues in ubiquitin that contribute to the formation of polyubiquitin chains on protein substrates, including K6, K11, K27, K29, K33, K48, and K63.^[^ ^26^ ^]^ Among them, lysine 48 (K48)‐linked polyubiquitination and lysine 63 (K63)‐linked polyubiquitination are the two most abundant types. Co‐immunoprecipitation (Co‐IP) experiments confirmed that Mei facilitated the ubiquitination of PKMYT1 and that the Mei‐induced polyubiquitination of PKMYT1 was completely terminated by K48‐mutant ubiquitin (K48R Ub) (Figure 4h). Conversely, K63‐mutant ubiquitin (K63R Ub) had no significant effect on PKMYT1 polyubiquitination (Figure S5e, Supporting Information). Therefore, K48‐linked polyubiquitinated ligation is the predominant ubiquitinated form for Mei‐induced PKMYT1 degradation.

### 2.5 Mei Serves as a Molecular Glue That Promotes PKMYT1‐TRIM25 Interaction and Facilitates PKMYT1 Degradation

UPS‐mediated protein degradation, which involves ubiquitin, ubiquitin‐activating enzyme (E1), ubiquitin‐binding enzyme (E2), ubiquitin‐protein ligase (E3), 26s proteasome, and deubiquitinating enzymes (DUBs), is a complex biological process.^[^ ^27^ ^]^ In this process, ubiquitin is activated by E1, then moved to E2, and the activated ubiquitin is transferred to the substrate protein mediated by the E3 ligase, which plays a great role in determining substrate specificity.^[^ ^28^ ^]^ Once the substrate protein is ubiquitinated, labeled protein will be recognized by the 26s proteasome for degradation.

**Figure 5:**
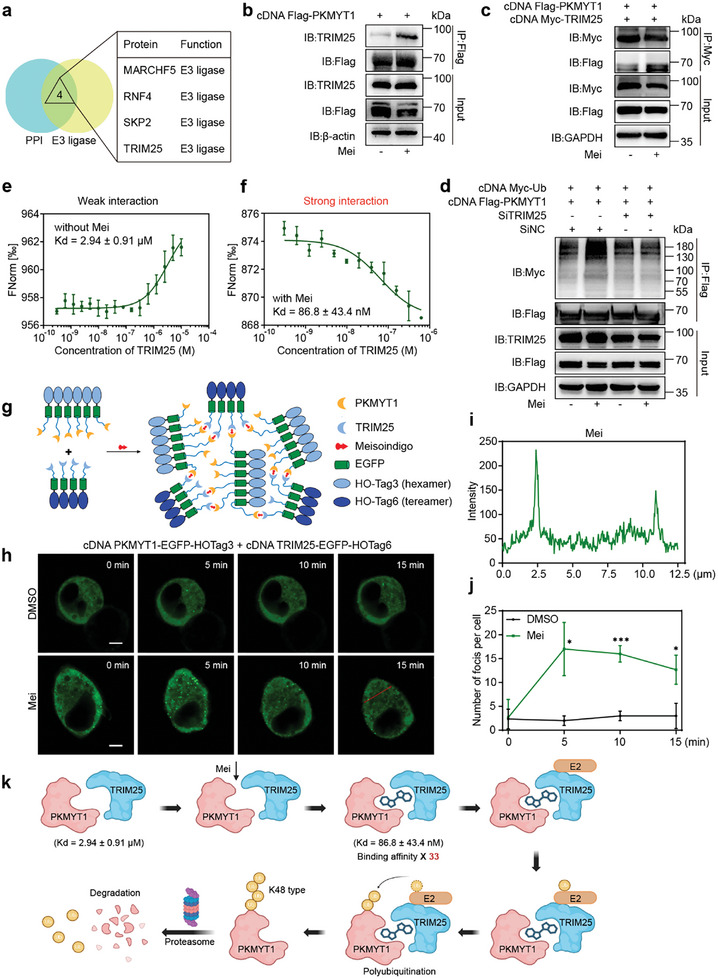
Mei serves as a molecular glue to enhance the PKMYT1‐TRIM25 interaction and promote PKMYT1 degradation. a) Venn diagram depicting the intersection between the protein‐protein interaction (PPI) network of PKMYT1 and the E3 ligase family. b) Co‐IP assays reveal interactions between PKMYT1 and E3 ligase TRIM25 in HEK293T cells. HEK293T cells overexpressing Flag‐PKMYT1 were treated with Mei (10 µM) or DMSO for 3 h. c) The interaction between TRIM25 and PKMYT1 was assessed via Co‐IP assay. HEK293T cells overexpressing Flag‐PKMYT1 and Myc‐TRIM25 were treated with Mei (10 µM) or DMSO for 3 h. d) Mei promoted the ubiquitination of PKMYT1 through the E3 ubiquitin ligase TRIM25. HEK293T cells were cotransfected with the indicated plasmids and siRNAs for 36 h and then treated with DMSO or Mei (10 µM) for 3 h. e) MST assay of TRIM25 binding to the recombinant PKMYT1 (75‐362) protein (n = 3). f) MST assay of TRIM25 binding to the recombinant PKMYT1 (75‐362) protein preincubated with Mei (n = 3). g) Schematic diagram showing the design of SPPIER experiment. h) EGFP‐fluorescence images of HEK293T cells expressing PKMYT1‐EGFP‐HOTag3 and TRIM25‐EGFP‐HOTag6 at the indicated time point after treatment with Mei (15 µM) or equivalent volume of DMSO (Scale bars = 5 µm). i) Fluorescence intensity analysis of the red line across the HEK293T cells treated with Mei (15 µM). j) Analysis of PKMYT1‐Mei‐TRIM25 ternary complex formation in HEK293T cells by quantifying the fluorescent foci numbers (n = 3). k) Schematic diagram showing how Mei acts as a molecular glue to enhance the interaction between PKMYT1 and TRIM25, triggering the degradation of PKMYT1 via the ubiquitin‐proteasome pathway. The data are presented as the means ± SDs. Statistical significance was assessed via two‐tailed unpaired Student's t‐test, *P < 0.05, **P < 0.01, ***P < 0.001 versus the control group. Figure (k) created with BioRender.com.

To identify the primary E3 ligase involved in PKMYT1 proteolysis, we cross‐analyzed the PKMYT1 interactome with an E3 ligase library, and identified TRIM25, MARCHF5, RNF4, and SKP2 as candidate ligases (**Figure** **5**a). Co‐IP assays in HEK293T cells overexpressing Flag‐PKMYT1 validated the interactions of these E3 ligases with PKMYT1, with TRIM25 displaying enhanced binding in the presence of Mei (10 µM) (Figure 5b; Figure S7a–c, Supporting Information). This finding was corroborated by an additional Co‐IP experiment in HEK293T cells co‐expressing Flag‐PKMYT1 and Myc‐TRIM25 (Figure 5c). MST experiments confirmed the binding affinity between Mei and recombinant TRIM25, with a *Kd* value of 0.22 ± 0.10 µM (Figure S7d, Supporting Information). To verify the role of TRIM25 in PKMYT1 protein degradation, we knocked down TRIM25 in HEK293T cells, which partially attenuated Mei‐induced PKMYT1 degradation (Figure S7e, Supporting Information). Furthermore, Mei‐induced polyubiquitination of PKMYT1 was significantly reduced following TRIM25 knockdown via small interfering RNA (siRNA) (Figure 5d). These results indicate that the E3 ligase TRIM25 plays a crucial role in the polyubiquitination and proteasomal degradation of PKMYT1.

To confirm the formation of the dynamic E3 ligase complex in vitro, MST assays were conducted to assess the binding affinity between TRIM25 and recombinant PKMYT1(75‐362) in the presence or absence of Mei. The MST assay revealed a relatively weak interaction between TRIM25 and PKMYT1 (*Kd* = 2.94 ± 0.91 µM), which markedly intensified (by 33‐fold, with *Kd* = 86.8 ± 43.4 nM) when PKMYT1 was preincubated with Mei (Figure 5e,f). Additionally, the separation of phases‐based protein interaction reporter (SPPIER) assay was used to confirm the trimer complex formation of PKMYT1‐Mei‐TRIM25 in living cells. The design of SPPIER assay is shown in Figure 5g, SPPIER protein was composed of three domains, including a protein‐of‐interest domain, an enhanced GFP (EGFP) domain, and a homo‐oligomeric tag (HOTag) domain.^[^ ^29^ ^]^ Upon small molecule‐induced PPI between proteins A and B, A‐EGFP‐HOTag3 protein hexamers crosslink B‐EGFP‐HOTag6 tetramers to induce EGFP separation, forming highly fluorescent green EGFP droplets.^[^ ^29^, ^30^ ^]^ In the SPPIER assay, live cell fluorescence imaging showed that treatment with Mei resulted in the formation of more green fluorescent puncta, compared to the control group (Figure 5h,j). This illustrates the formation of a PKMYT1‐Mei‐TRIM25 trimer complex in live cells.

In contrast, when the cysteine residue at position 301 of PKMYT1 was mutated, MST experiments showed that Mei failed to enhance the binding affinity between PKMYT1 (75‐362) C301A and TRIM25 (Figure S8a,b, Supporting Information). Additionally, in HEK293T cells expressing Flag‐PKMYT1 (C301A) mutant, Co‐IP assays further confirmed that Mei could not augment the binding between PKMYT1 (C301A) and TRIM25 (Figure S8c, Supporting Information), nor could it increase the ubiquitination level of PKMYT1 (C301A) (Figure S8d, Supporting Information).

Collectively, these findings establish Mei as a molecular glue that covalently binds to Cys301 of PKMYT1 and promotes the assembly of the PKMYT1‐TRIM25 complex, leading to PKMYT1 ubiquitination and subsequent proteasomal degradation (Figure 5k).

### 2.6 PKMYT1 Knockdown Inhibits Cell Proliferation and Promotes Mitochondrial Dysfunction

**Figure 6:**
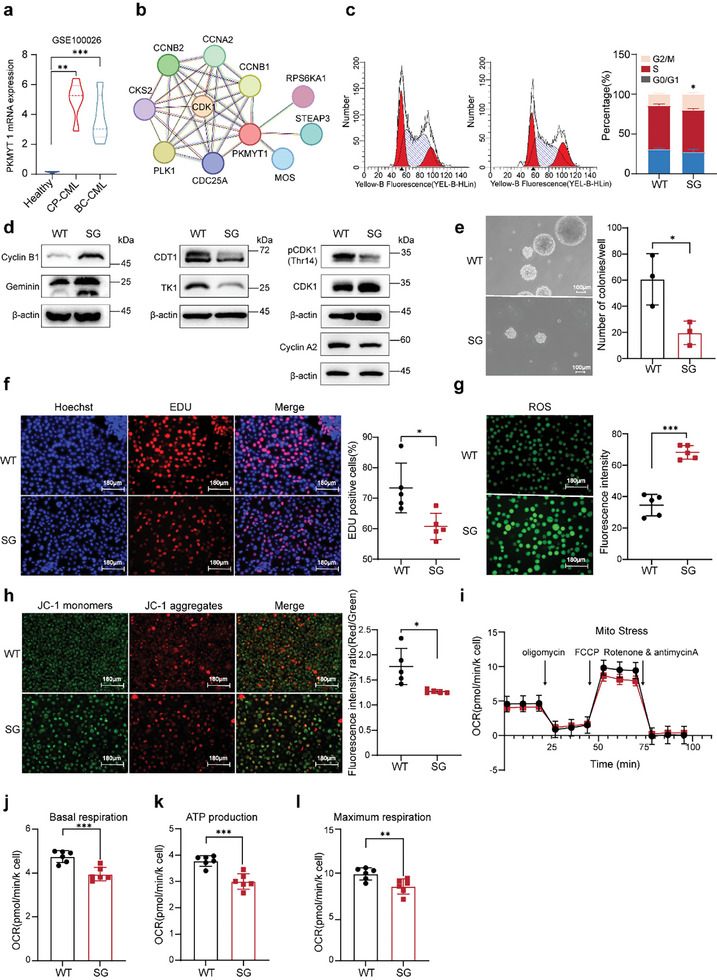
PKMYT1 knockdown inhibits K562 cell growth. a) The mRNA expression of PKMYT1 in peripheral blood from the GSE100026 database in healthy individuals, patients with chronic myeloid leukemia (CML) in the chronic phase, and patients with CML in the blast crisis. b) Key genes linked to PKMYT1 in the STRING database. c) The effect of PKMYT1 knockdown on G2/M cell cycle transition in K562 cells was assessed by flow cytometry (n  =  3). d) The effects of PKMYT1 knockdown on cell cycle proteins in K562 cells were examined by immunoblotting. e) Detection of the effect of PKMYT1 knockdown on K562 cell proliferation via a soft agar colony formation assay (n  =  3). f) Detection of the effect of PKMYT1 knockdown on K562 cell proliferation via EdU staining (n = 5). g) Effect of PKMYT1 knockdown on ROS levels in K562 cells (n = 5). h) Effect of PKMYT1 knockdown on the mitochondrial membrane potential in K562 cells (n = 5). i) Oxygen consumption rate (OCR) levels in wild‐type or PKMYT1‐knockdown cells were assessed using a Seahorse XF24 analyzer (n  =  6). j‐l), Basal respiration, ATP production, and Maximum respiration were assessed (n  =  6). For 6c,e‐h,j‐l, the data are presented as the means ± SDs. Statistical significance was assessed via two‐tailed unpaired Student's t‐test, *P < 0.05, **P < 0.01, ***P < 0.001 versus the control group.

We further surveyed the mRNA expression of PKMYT1 in CML patients across different disease stages by searching data from National Center for Biotechnology Information (NCBI). Compared with those in healthy controls, PKMYT1 mRNA levels were elevated in both chronic‐phase leukemia patients and blast leukemia patients (**Figure** **6**a). A PPI network constructed using the Search Tool for the Retrieval of Interacting Genes (STRING) database revealed associations between PKMYT1 and genes involved in cell cycle regulation, including CCNB1, CCNB2, CDK1, CCNA2, and CDC25A (Figure 6b), which is consistent with previous literature.^[^ ^31^ ^]^ Additionally, flow cytometry analysis of K562 cells revealed that PKMYT1 knockdown increased the proportion of cells in the G2/M phase (Figure 6c), which is consistent with the observation of Mei‐induced G2/M arrest in HT‐29 cells.^[^ ^32^ ^]^ Following PKMYT1 knockdown, reduced expression of CDT1, thymidine kinase 1 (TK1), phosphorylation of CDK1 at Thr14 and Cyclin A2 was observed, whereas CyclinB1, Geminin, and CDK1 expression increased (Figure 6d). 5‐Ethynyl‐2′‐deoxyuridine (EdU) staining and soft agar colony formation assays revealed significantly less proliferation in PKMYT1‐knockdown K562 cells than in wild‐type K562 cells (Figure 6e,f). In addition, Mei treatment inhibited the proliferation and colony formation ability of K562 cells in a dose‐dependent manner (Figure S9a,b, Supporting Information). PKMYT1 knockdown elevated ROS levels (Figure 6g), indicative of oxidative stress, accompanied by early apoptotic shifts, as evidenced by the predominance of green fluorescence in the JC‐1 assay (Figure 6h). Treatment with Mei induced comparable effects, consistent with the role of PKMYT1 in modulating cellular redox homeostasis and apoptotic pathways (Figure S9c,d, Supporting Information). Unlike most solid tumor cells, CML is initiated and maintained by self‐renewing malignant CD34^+^ stem cells, which exhibit higher oxygen consumption rates (OCRs) than normal CD34^+^ hematopoietic cells do.^[^ ^33^ ^]^ Seahorse analysis revealed that, compared to wild‐type K562 cells, PKMYT1‐knockdown cells presented lower OCRs, basal respiration, ATP production, and maximal respiration (Figure 6i‐l). Collectively, these findings underscore the pathological significance of PKMYT1 in CML and its potential as a therapeutic target for inhibiting tumor cell proliferation and promoting mitochondrial dysfunction.

### 2.7 PKMYT1 Knockdown Inhibits Leukemia Cell Proliferation and Delays Leukemia Progression In Vivo

**Figure 7:**
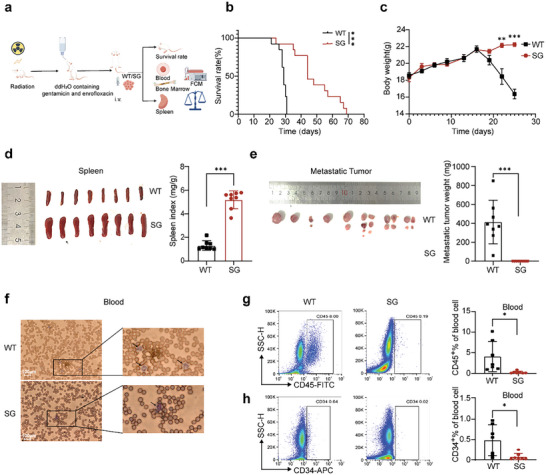
PKMYT1 knockdown inhibits leukemia cell proliferation and delays leukemia progression in vivo. a) Flowchart of the chronic myeloid leukemia orthotopic xenograft model. b) Survival of mice inoculated with wild‐type K562 and PKMYT1‐knockdown K562 cells (n  =  13). c) The body weights of the mice inoculated with wild‐type K562 or PKMYT1‐knockdown K562 cells were measured every 3–4 days (n  =  8). d) Spleen image and spleen indices of PKMYT1‐knockdown and wild‐type group mice (n  =  8). e) Image and weights of metastatic tumors from PKMYT1‐knockdown and wild‐type group mice (n  =  8). f) Blood smear results for PKMYT1‐knockdown and wild‐type group mice (n = 8). g‐h) Human CD45^+^ and CD34^+^ cell content in the peripheral blood of PKMYT1‐knockdown and wild‐type group mice (n  =  7). For 7e, narrow spacing between metastatic tumors indicates that these tumors originate from the same mouse, whereas wide spacing suggests that the tumors originate from different mice. For 7c, the data are presented as the means ± SEMs; for 7d,e,g,h, the data are presented as the means ± SDs. Statistical significance was assessed via two‐tailed unpaired Student's t‐test, *P < 0.05, **P < 0.01, ***P < 0.001 versus the wild‐type group. Figure a created with figdraw.com.

To further define the role of PKMYT1 in regulating CML progression in vivo, an orthotopic xenograft model was established via intravenous (i.v.) injection of K562 tumor cells. Specifically, after receiving 1 Gy irradiation, NSG mice were randomly divided into two groups and injected with 1 × 10^7^ PKMYT1‐knockdown K562 cells or wild‐type K562 cells. The mice were given free access to pure water containing gentamicin and enrofloxacin, and the state of the animals and the course of the disease were observed (**Figure** **7**a). Consistent with the in vitro results, PKMYT1 knockdown significantly delayed tumor progression in vivo. All the mice inoculated with wild‐type K562 cells died within two weeks after disease onset, whereas those injected with PKMYT1‐knockdown K562 cells presented delayed leukemia onset and slower leukemia progression, with a survival time of up to 40 days from onset (Figure 7b).

The mice that received wild‐type K562 cells displayed depression, piloerection, a hunched posture, and significant weight loss, whereas the mice received PKMYT1‐knockdown K562 cells maintained stable weights and good activity levels (Figure 7c). Compared with those inoculated with wild‐type cells, those inoculated with PKMYT1‐knockdown cells presented increased spleen and liver indices (Figure 7d; Figure S10a, Supporting Information).

All the mice injected with wild‐type K562 cells developed distant metastases in mesenteric, axillary, and inguinal lymph nodes by day 25 post‐inoculation, whereas no metastases were observed in the mice received PKMYT1‐knockdown K562 cells (Figure 7e). Furthermore, the mice of wild‐type group presented increased numbers of myeloid cells in the peripheral blood and bone marrow, whereas the mice of PKMYT1‐knockdown group presented predominantly erythrocytes in the peripheral blood, with fewer myeloid cells and more erythrocytes in the bone marrow (Figure 7f; Figure S10b, Supporting Information). Flow cytometry revealed a significant reduction in the percentage of CD45^+^ cancer cells in the peripheral blood of the mice received PKMYT1‐knockdown K562 cells, compared with that in the peripheral blood of the mice of wild‐type group (Figure 7g). Similar results were observed in the bone marrow (Figure S10c, Supporting Information). Additionally, the percentage of CD34^+^ cancer stem cells was decreased in the peripheral blood of the mice of PKMYT1‐knockdown group (Figure 7h). These findings highlight the critical role of PKMYT1 in leukemia cell pathogenesis and metastasis, as well as the potential therapeutic benefits of inhibiting PKMYT1 in vivo.

## 3 Discussion

The identification of cellular targets for small molecules, particularly those with an established clinical history, is pivotal for advancing our understanding of their therapeutic mechanisms and optimizing their clinical utility.^[^ ^23^, ^34^ ^]^ Mei is a drug that has been used in the clinic for more than 30 years for the treatment of CML, yet its definitive molecular target remains elusive.^[^ ^20c,d^ ^]^ In this study, we have identified the primary cellular target of Mei via ABPP and demonstrated that Mei binds covalently and reversibly to the Cys301 residue of PKMYT1. Notably, Mei acts as a unique natural molecular glue, increasing the binding affinity between PKMYT1 and the E3 ligase TRIM25 33‐fold, thereby promoting the K48‐linked ubiquitination and degradation of PKMYT1, leading to therapeutic effects against CML. Additionally, the reversible covalent reaction between Mei and cysteine provides new insights for the development of reversible reactive groups in drug discovery.

In recent years, targeted protein degradation (TPD) strategies, including molecular glues, PROTACs, and lysosome‐mediated TPD, have emerged as novel therapeutic approaches for inducing the degradation of “undruggable” proteins.^[^ ^13^ ^]^ In terms of drugs targeting PKMYT1, the current sole clinical candidate, RP‐6306, is a selective inhibitor in phase II clinical trials for the treatment of various solid tumors.^[^ ^35^ ^]^ There are often challenges in the use of kinase inhibitors; these include off‐target effects and tumor resistance, which increase the likelihood of treatment failure.^[^ ^36^ ^]^ In contrast, degradants offer the potential to overcome these issues at the mechanistic level. Mei functions as a molecular glue capable of selectively targeting PKMYT1 for degradation, providing an important biochemical foundation for the development of PKMYT1 degraders. Specifically, Mei‐based molecular glues and Mei‐derived PROTACs, such as CRBN‐type and VHL‐type PROTACs, can be designed and synthesized to enhance the degradation of PKMYT1, thereby exhibiting more potent tumor suppressive effects.

As a key member of the Wee family, PKMYT1 plays an important role in the cell cycle progression of mitosis as well as in the occurrence, progression, metastasis, and poor prognosis of various solid tumors.^[^ ^2^, ^4^, ^5^ ^]^ Despite the frequent overexpression of PKMYT1 and its established synthetic lethal relationship with CCNE1 amplification,^[^ ^37^ ^]^ its role in CML remains understudied. In this study, we report for the first time that PKMYT1 plays a significant role in CML progression. Knocking down PKMYT1 significantly slowed tumor growth in both ectopic and orthotopic xenograft models and prolonged the survival period in a leukemia mouse model. PKMYT1 was also identified as a negative regulator of cell cycle transition that inhibits CDK1‐Cyclin B complex formation by phosphorylating Tyr14/Tyr15. Recent studies have shown that PKMYT1 expression is increased in hepatocellular carcinoma (HCC), non‐small cell lung cancer (NSCLC), and pancreatic ductal adenocarcinoma (PDAC)^[^ ^5^, ^38^ ^]^ and is associated with poor survival probability. To reveal the role of PKMYT1 in leukemia, we identified PKMYT1 as an oncogenic factor that promotes CML progression using data sets from multiple web‐based sources. Given the function of PKMYT1, we have shown that the deletion of PKMYT1 in K562 cells induces G2/M phase arrest, regulates the expression of cell cycle‐related proteins, induces mitochondrial damage, and inhibits cell proliferation. These findings suggest that PKMYT1 plays a critical role in the progression of CML.

CML progresses through distinct stages, namely, the chronic, accelerated, and blast stages, each of which poses unique challenges. During the chronic phase, patients typically respond favorably to treatment. However, transitioning into the accelerated phase marks a turning point where tumor cells become more aggressive, accelerating cancer progression and posing a grave threat to patients' lives.^[^ ^39^ ^]^ Our results demonstrated that knocking down PKMYT1 in cancer cells delayed CML onset in mice and significantly slowed cancer progression. Notably, we found that PKMYT1 knockdown significantly suppressed leukemic cell metastasis to lymph nodes and markedly decreased the number of leukemic stem cells, which may be linked to the ability of PKMYT1 to modulate Wnt signaling pathway activity,^[^ ^38a^ ^]^ thereby regulating cancer stem cell function. Our findings suggest that PKMYT1 may be a potential therapeutic target for CML treatment. Furthermore, we conducted some preliminary cytotoxic activity and discovered that RP6306, a specific inhibitor of PKMYT1, exhibited substantial cytotoxic effects against some solid tumor cell lines, hinting that PKMYT1 could potentially serve as a therapeutic target not only for CML, but also for solid tumors.

## 4 Experimental Section

### Synthesis and Physical Data Assay

See the Supporting Information for details.

### Cell Culture, Cytotoxicity Assay, and Cell Proliferation Assay

The human chronic myeloid leukemia cell line K562 was purchased from Procell (Wuhan, China). HEK293T, MOLM‐13, MV‐4‐11, Jurkat, Romas, DOHH2, Pfeiffer, and OCI‐LY3 cells were purchased from the Peking Union Medical College Cell Bank (Beijing, China). HEK293T cells were cultured in DMEM supplemented with 10% FBS at 37 °C with 5% CO_2_. The other cell lines were cultured in RPMI‐1640 medium supplemented with 10% FBS at 37 °C with 5% CO_2_. DMEM, RPMI‐1640 medium, PBS, and 0.25% trypsin‐EDTA were purchased from Solarbio (Beijing, China). FBS was purchased from Procell (Wuhan, China).

Cell Counting Kit‐8 (CCK‐8) was used for the cytotoxicity and proliferation assays. For the cytotoxicity assay, cells (5 × 10^3^/well) were seeded into 96‐well culture plates for 6 h. Then, Mei or MP was added at a series of concentrations. After 48 h or 72 h of incubation, the CCK‐8 reagent was added, and the mixture was incubated for another 2 h. Then, the absorbance at 450 nm was measured with a microplate reader (Thermo Fisher Scientific). The data were normalized to those of the control group (0.1% DMSO). GraphPad Prism 8.0 was used to calculate the IC_50_ values. For the proliferation assay, cells (4 × 10^3^/well) were seeded into 24‐well culture plates, the CCK‐8 reagent was added every 48 h, and the plates were incubated for another hour. The absorbance was measured, and the proliferation curves were plotted with GraphPad Prism 8.0.

### In Situ and In Vitro Gel‐Based Fluorescence Labeling and Coomassie Blue Staining

The fluorescence labeling experiment was performed as reported previously.^[^ ^40^ ^]^ For the dose‐dependent fluorescence labeling experiments, the cells were seeded in 6‐well culture plates, treated with MP (0–40 µM), and incubated at 37 °C with 5% CO_2_ in an incubator for 1 h. The cells were collected, washed with PBS 3 times, lysed in 100 µL of 0.2% NP‐40/PBS buffer, and repeatedly frozen and thawed three times to promote cell lysis. The resulting lysates were centrifuged at 14 000 rpm for 30 min to remove cell debris. The protein concentration of each sample was quantified with a Pierce BCA protein assay kit (Thermo Fisher Scientific) and adjusted to 1 mg mL^−1^. Click reaction of each protein sample was performed by adding final concentrations of 100 µM rhodamine‐N3, 100 µM tris(benzyltriazolylmethyl)amine (TBTA), 1 mM tris (2‐carboxyethyl) phosphine (TECP), and 1 mM CuSO_4_, followed by incubation at 37 °C for 1 h. The samples were subsequently centrifuged at 14 000 rpm for 10 min at 4 °C to collect the protein pellet. Then, the protein pellets were washed with cool methanol 3 times, swept dry, dissolved in 40 µL of 1% SDS/PBS, and boiled with 10 µL of 5 × SDS‐PAGE loading buffer. The samples were separated via 10% SDS‐PAGE. The gels were imaged via a rhodamine channel on a Typhoon FLA 9500 and stained with Coomassie blue (CBB) to indicate equal sample loading.

For the time‐dependent fluorescence labeling experiments, the cells were treated with MP (5 µM) and incubated at 37 °C with 5% CO_2_ in an incubator for 1–4 h. Subsequent experiments were performed in accordance with the protocol above.

For the competition fluorescence labeling experiments, the cells were pretreated with or without Mei (10, 25, or 50 µM) for 30 min, treated with MP (5 µM) for another hour, and incubated at 37 °C with 5% CO_2_ in an incubator. Subsequent experiments were performed in accordance with the protocol above.

### In Situ Pull‐Down Experiment and Target Validation

The in situ pull‐down experiment was performed and adapted from previous reports.^[^ ^41^ ^]^ Briefly, K562 cells were harvested, resuspended in fresh medium, and incubated with DMSO or MP (5 µM) at 37 °C with 5% CO_2_ in an incubator for 1 h. After incubation, the cells were harvested, washed twice with PBS, and lysed in 1 mL of 0.2% NP‐40/PBS. The resulting lysates were centrifuged at 14 000 rpm for 30 min to remove cell debris, after which the protein concentrations were determined by using a Pierce BCA protein assay kit and normalized to 2 mg mL^−1^ in a volume of 1 mL. Cell lysates were incubated with 100 µM biotin‐N3, 100 µM TBTA, 1 mM TECP, and 1 mM CuSO_4_, at 37 °C for 1 h. After the click reaction, the proteins were precipitated by adding 5 mL of methanol at −20 °C for 8 h. The protein precipitates were collected by centrifugation (8000 ×g, 5 min) and washed three times with cold methanol, then resuspended and dissolved in 1 mL of 1.2% SDS/PBS. The proteins were boiled at 90 °C for 5 min and after centrifugation at 1400×g for 1 min at room temperature, the supernatant was diluted to 0.2% SDS/PBS. The above supernatant was enriched with 100 µL of streptavidin agarose beads (Thermo Fisher Scientific) for 4 h at room temperature. Notably, the beads need to be pre‐washed three times with PBS before the experiment. After 4 h incubation, the protein‐enriched beads were washed three times with 5 mL PBS and three times with 5 mL H_2_O, and then resuspended in 500 µL of 6 M urea/PBS. After reduction with 10 mM dithiothreitol (DTT) at 37 °C for 60 min, alkylation was performed with 20 mM iodoacetamide (IAA) at 35 °C for 30 min in the dark.

For the label‐free quantitative proteomics experiment, the beads were washed twice with 1 mL of H_2_O. The enriched proteins were digested by 2 µg of trypsin (Promega) in 200 µL of 2 M urea/PBS with 2 µL of 100 mM CaCl_2_ for 16 h at 37 °C. The digested peptide samples were collected and desalted with C18 Tips (Thermo Fisher Scientific) before LC‐MS/MS analysis.

For the TMT‐labeled quantitative proteomics experiment, the beads were washed 7 times with 1 mL of 100 mM triethylammonium bicarbonate buffer (TEAB), resuspended in 200 µL of 100 mM TEAB, and then 2 µg of trypsin was added. The resulting mixture was incubated at 37 °C for 16h. TMT reagents (Thermo Scientific) were used for peptide labeling, and 42 µL of anhydrous acetonitrile was added to dissolve 0.8 mg of TMT. Subsequently, 10 µL of the TMT solution was added to the samples, which were then incubated at 20 °C with shaking at 600 rpm for 1 h. After incubation, 8 µL of 5% hydroxylamine was added to quench the reaction. Finally, the labeled peptides were mixed together and dried via vacuum centrifugation. Peptide fractionation was conducted on an ACQUITY Arc Bio system (Waters) equipped with a Waters Bridge column (3.5 µm, 2.1×150 mm). Mobile phase A was MeCN‐H_2_O (2:98, 0.1% ammonia), while mobile phase B was MeCN‐H_2_O (98:2, 0.1% ammonia). The 51 min gradient procedure was as follows: 0% B for 10 min, 0–5% B for 0.1 min, 5–8% B for 1.9 min, 8–16% B for 11 min, 16–32% B for 21 min, 32–95% B for 1 min, 95% B for 1 min, 95–15% B for 2 min, and 15% B for 3 min, 400 µL min^−1^, 214 nm. The fractions were dried, reconstituted in 1% formic acid (FA) in ddH_2_O, centrifuged (15000 rpm, 4 °C, 30 min), and subjected to LC‐MS/MS analysis.

The peptides in the quantitative proteomics experiments were detected via an Ultimate 3000 Nano HPLC instrument coupled with an Orbitrap Fusion Lumos Tribrid mass spectrometer (Thermo Fisher Scientific). The LC column was an analytical column (Acclaim PepMap C18 75 µm × 15 cm). Mobile phase A was water with 0.1% FA, and mobile phase B was 80% MeCN with 0.1% FA. The gradient was as follows: 4% B for 5 min; 4%‐30% B for 65 min; 30–80% B for 5 min; 80% B for 5 min; 80–4% B for 5 min; and 4% B for 5 min, 300 nL min^−1^. The peptides were ionized by using a spray voltage of 2.4 kV and a capillary temperature of 320 °C. The instrument was operated in data‐dependent mode, automatically switching between MS and MS2 scans. For the full proteome samples, full‐scan MS spectra (m/z 350–1500) were acquired with a maximum injection time of 50 ms at 60 000 resolution and an automatic gain control (AGC) target value of 4 × 10^5^ charges.

### Peptide/Protein Identification and Quantification

In the ABPP experiment, label‐free quantitative proteomics LC‐MS/MS data analysis was performed using MaxQuant software (version 1.6.5.0), and the resulting data were further analyzed using the Perseus software (version 1.6.15.0). TMT‐labeled quantitative proteomics LC‐MS/MS data were analyzed using the Proteome Discoverer (2.3) workstation. For the binding site experiments, peptide identification was performed using the Peaks Studio 11 software and default settings with static modification of cysteine (+57.0215 Da), variable oxidation of methionine (+15.9949 Da), and variable Mei (+276.0899 Da).

### The Recovery Rate Measurement of Mei Dissociates from Recombinant PKMYT1 (75‐362) Protein Upon Unfolding

The recovery rate measurement of Mei was measured and adapted from previous reports.^[^ ^42^ ^]^ Mei dissociates from the recombinant PKMYT1 (75‐362) protein upon unfolding. To a solution of Mei in 50 µL PBS (8 µM) was added 50 µL of recombinant PKMYT1 (75‐362) (16 µM, 2.0 equiv) in PBS. After 1 h at 37 °C, 100 µL of 6 M guanidine (pH 7.4) was added, and the reaction mixture was incubated for different durations at 37 °C to unfold recombinant PKMYT1 (75‐362). At the indicated time points, MeCN (200 µL) was added, and the recovery of free Mei was quantified via LC‐MS/MS relative to a control sample containing Mei but lacking recombinant PKMYT1 (75‐362).

### The Decomposition Curve Measurement of Mei‐BME

The decomposition curve was measured as previously reported.^[^ ^42^ ^]^ A solution of thioether Mei‐BME in DMSO (1 mM) was diluted 100‐fold into PBS, pH 7.4, to afford a 10 µM solution. Aliquots of the solution were quenched by the addition of an equal volume of 4% FA in MeCN, prior to immediate and subsequent LC‐MS/MS analysis. The amount of adduct remaining at each time point was quantified, and the apparent first‐order rate constant was determined by fitting the data to a first‐order decay curve using GraphPad Prism 8.0.

### Fluorescence Labeling and Coomassie Blue Staining Experiments of the Recombinant Wild‐Type and Mutant PKMYT1 Proteins

The concentrations of the recombinant wild‐type and mutant PKMYT1 proteins were determined using a Pierce BCA protein assay kit and normalized to 0.2 mg mL^−1^. About 50 µL of protein sample was treated with MP (pretreated with or without GSH) at the indicated concentration and incubated at 37 °C for 1 h, after which final concentrations of 100 µM rhodamine‐N3, 100 µM TBTA, 1 mM TECP, and 1 mM CuSO_4_ were added, and the mixture was incubated at 37 °C for another hour. 500 µL of cold methanol was used to precipitate the proteins. The protein pellet was harvested by centrifugation at 14 000 rpm for 10 min at 4 °C, washed with cool methanol three times, swept dry, and dissolved. The samples were subjected to SDS‐PAGE fluorescence detection and CBB detection.

### CETSA

CETSAs were conducted as reported previously.^[^ ^43^ ^]^ For the temperature‐dependent CETSA, K562 cells were treated with DMSO or Mei (10 µM) and incubated for 1 h. Then, the cells were collected and resuspended in PBS. The cell suspension was divided into 11 aliquots, and each aliquot was heated at the indicated temperatures for 3 min on a PCR instrument (Bio‐Rad), and kept at room temperature for 3 min. The samples were subsequently frozen in liquid nitrogen three times and centrifuged at 14 000 rpm at 4 °C for 30 min. The supernatants were subsequently used for immunoblotting analysis of PKMYT1.

For the dose‐dependent CETSA, K562 cells were harvested and washed twice in PBS. Then the cells were lysed in 1 mL of 0.2% NP‐40/PBS and frozen in liquid nitrogen three times. The resulting lysates were centrifuged at 14 000 rpm for 30 min to remove cell debris. A total of 70 µL of each cell lysate (1 mg mL^−1^) was incubated with the indicated concentration of Mei (0–20 µM) at 37 °C for 30 min, heated at 47 °C for 3 min, and kept at room temperature for 3 min. The supernatants were isolated by centrifugation and used for immunoblotting analysis of PKMYT1.

### Expression and Purification of Recombinant Wild‐Type PKMYT1 (75‐362) and Its Mutants

The plasmids expressing wild‐type PKMYT1 (residues 75–362) and mutants were composed of the pET‐28a (+) vector and fused to a thrombin‐cleavable N‐terminal 6×His tag. Recombinant wild‐type PKMYT1 protein and mutants were expressed in *E. coli* BL21(DE3). Briefly, the bacteria were grown at 37 °C in 40 mL of LB medium supplemented with 50 µg mL^−1^ kanamycin with shaking at 220 rpm min^−1^ overnight. The diluted culture consisted of 200 mL of LB medium containing 50 µg mL^−1^ kanamycin with shaking at 220 rpm min^−1^ until the optical density at 600 nm was 0.6. The temperature was subsequently lowered to 18 °C, IPTG was added to a final concentration of 0.5 mM, and the shaking speed was decreased to 160 rpm min^−1^. The culture was incubated for 18 h, and the bacterial pellet was harvested via centrifugation. The resulting bacterial pellet was suspended in 35 mL of lysis buffer (20 mM Tris‐HCl, 150 mM NaCl, pH7.5) and lysed by sonication for 30 min at 0 °C. The *E. coli* lysate was centrifuged at 4 °C, and 10 000 rpm for 30 min, the protein supernatant was loaded onto a Ni‐resin (Gen Script) column, and the bound protein was eluted using a gradient from 20 to 400 mM imidazole buffer. Fractions containing PKMYT1 were detected on the SDS‐PAGE gel and concentrated. The protein was finally stored in buffer (20 mM Tris‐HCl, 150 mM NaCl, and 10% glycerol, pH7.5).

### Microscale Thermophoresis Analysis

Purified proteins were labeled using the Monolith Protein Labeling Kit RED‐NHS 2nd Generation Kit (Nano Temper Technologies), incubated with or without Mei (50 µM) for 30 min, and then diluted with assay buffer (40 nM, 0.05% Tween‐20). Mei or TRIM25 proteins (Origene, TP303757) were gradient diluted 2‐fold and then mixed with the labeled proteins. The mixtures were incubated and then loaded into Monolith standard treated capillaries. Thermophoresis was detected using a Monolith NT.115 instrument (NanoTemper Technologies) and *Kd* values were calculated using MO. Affinity Analysis software v2.3. Interactions were tested in three replicate experiments.

### Determination of the Mei‐Binding Site on PKMYT1 via LC‐MS/MS

Recombinant PKMYT1 (residues 75−362) (30 µL, 10 µM) in PBS buffer (pH 7.4) was incubated with Mei (final concentration, 20 µM, 2.0 equiv) at 37 °C for 1 h. The sample was then added 150 mL of a solution of 8 M urea with 100 mM DTT and incubated at 37 °C for 1 h. The sample was then transferred to a 10 kDa cut‐off filter (Millipore). After centrifugation at 14000 rpm for 20 min, the sample was washed with 150 µL of 50 mM NH_4_HCO_3_ at 14 000 rpm for 20 min. Then 150 µL of 20 mM IAA was added to the filter and incubated for another hour at room temperature in the dark. After centrifugation at 14 000 rpm for 20 min, the sample was washed with 150 µL of 50 mM NH_4_HCO_3_ three times. Next, 150 µL of 30 mM NH_4_HCO_3_ and Glu‐C (Glu‐C: protein = 1:10, w/w) were added to the sample, followed by further incubation at 37 °C for 16 h. The digested peptides were collected and desalted for subsequent LC‐MS/MS analysis.

### Molecular Dynamics (MD) Simulation

The crystal structure of PKMYT1 (PDB ID: 5VCZ) was used to generate the initial covalent docking complex of PKMYT1‐Mei. The reactive covalent site for PKMYT1 is Cys301. Then the molecular dynamics simulations were carried out. All MD simulations were performed in GROMACS (version 2020.6).^[^ ^44^ ^]^ The AMBER ff99SB force field and general AMBER force field (GAFF) were used for modeling proteins and ligands, respectively. The bcc charge of the ligand was calculated with the aid of the ACPYPE script based on the Amber Tools 20 suite. The system was solvated in a cubic box with a TIP3P water model. The system was neutralized by adding a corresponding number of sodium or chloride (Na^+^/Cl^−^) ions. The solvent layers between the box edges and solute surface were set to 10 Å. The particle mesh Ewald (PME) method was employed to treat the long‐range electrostatic interactions and the cut‐off of van der Waals interactions was set to 10 Å. Then, a minimization step was performed on the system via the conjugate gradient method to eliminate any unreasonable contacts between atoms. Next, a short NPT equilibration was carried out for 0.01 ns in a 1 fs time step. For the equilibration phase, the temperature was controlled via the V‐rescale thermostat algorithm with a time constant of 0.2 ps. The pressure was controlled via the Berendsen coupling algorithm with a time constant of 0.5 ps. Finally, production simulation was conducted with the time step set as 1 fs. The temperature of the simulation systems was controlled and kept constant by the V‐rescale thermostat algorithm and the Berendsen coupling algorithm was used to keep the pressure constant. The simulated temperature was set to 300 K, and the pressure was set to 101.3 kPa. The molecular dynamics simulation was performed in the NPT ensemble within 200 ns. The binding free energy of the protein with the ligand was calculated using gmx_MMPBSA (version 1.4.3).

### RNA Isolation and Quantitative RT‐PCR

The K562 cells were harvested and total RNA was extracted by using TransZol Up Plus RNA Kit (TransGen Biotech). The mRNA was reverse transcribed into cDNA by using TransScript One‐Step gDNA Removal and cDNA Synthesis Super Mix (TransGen Biotech). RT‐PCR was performed using PerfectStart Green qPCR SuperMix (TransGen Biotech) on a QuantStudio 3 system (Thermo Fisher Scientific). Real‐time PCR amplification was then performed as 45 cycles of 94 °C for 5 s, and 60 °C for 30 s. The threshold cycle (CT) values were finally provided. And the relative transcriptional level of PKMYT1 gene normalized to GAPDH was calculated by the comparative 2^−ΔΔ^ *^Ct^*. The sequences of the primers used for quantitative PCR are listed in Table S1 (Supporting Information).

### Plasmids and Transfections

The generation of the PKMYT1 plasmid for mammalian expression, in which the cDNA was subcloned and inserted into pcDNA3.1(+) containing a Flag tag at the N‐terminal region, was performed by Genomeditech (Shanghai, China). The generation of the Ub plasmid or mutants and the TRIM25 plasmid for mammalian expression, in which the cDNA was subcloned and inserted into pcDNA3.1(+) containing a Myc tag at the N‐terminal region, was performed by BGI.Write (Beijing, China). To create PKMYT1‐EGFP‐HOTag3 fusions, the target protein was cloned into pcDNA3.1 containing EGFP. HOTag3 was then cloned into the pcDNA3.1 PKMYT1‐EGFP construct, resulting in pcDNA3.1 PKMYT1‐EGFP‐HOTag3. Similar procedures were performed to produce pcDNA3.1 TRIM25 ligase‐EGFP‐HOTag6. PKMYT1‐EGFP‐HOTag3, TRIM25 ligase‐EGFP‐HOTag6, and Flag‐PKMYT1 (C301A) mutant plasmids were performed by BGI.Write (Beijing, China).

HEK293T cells were transiently transfected with the above plasmids using Lipofectamine 3000 Transfection Reagent (Invitrogen, L3000015) and Opti‐MEM (Gibco, 31 985 062) for 24 h, according to the manufacturer's instructions.

siRNAs for gene knockdown were purchased from Sangon Biotech (Shanghai, China). Transfection was performed with Lipofectamine RNAiMAX Reagent (Invitrogen, 13 778 150) and Opti‐MEM according to the manufacturer's instructions. Cells were harvested 36 h after transfection. The sequence of the siRNA used was as follows: siTRIM25: CCUCGACAAGGAAGAUAAATT.

### Co‐immunoprecipitation (Co‐IP) assay

HEK293T cells were transiently transfected with Flag‐PKMYT1 and Ub (wild‐type Ub and K48R or K63R Ub) plasmid DNA and cultured in an incubator. After 24 h, the cells were treated with Mei (10 µM), incubated for another 3 h and washed twice with PBS. The cells were then harvested and lysed in Western and IP cell lysates (Beyotime) for 30 min. The cell lysates were centrifuged and the supernatant was incubated with 30 µL of DYKDDDDK‐Nanoab‐Agarose (LABLEAD) at 4 °C for 3 h. The agarose was harvested by centrifugation at 2500 xg at 4 °C for 3 min, washed 5 times with PBS, and boiled with SDS‐PAGE loading buffer. Finally, the bound proteins were detected by immunoblotting with the indicated antibodies.

### Generation of PKMYT1‐knockdown K562 cell lines by CRISPR/Cas9 system

The generation of PKMYT1‐knockdown K562 cell lines using the CRISPR/Cas9 system was performed by Cyagen (Suzhou, China). A chimeric guide RNA (5′‐CGTTCCATGTCACCATTCCG‐GGG‐3′) was introduced into the cells by electroporation. After electroporation, monoclonal cells were selected and verified by PCR and sequencing, and SG cell lines (heterozygous cells) with human PKMYT1 gene knockdown were successfully obtained. PKMYT1 knockdown was detected by Western blotting, and DNA sequencing using primers (forward: 5′‐ AGAGGAGTTTTCCCTTGTATGAGTC ‐3′, reverse: 5′‐ GAGGCAGGTTACACAAAGTGG ‐3′).

### Soft Agar Colony Formation Assay

About 2 mL of 0.6% agar in RPMI‐1640 medium, containing 10% FBS, was prewarmed to 45 °C, transferred to 6‐well plates, and stored at 25 °C to solidify the bottom agar. Wild‐type and PKMYT1‐knockdown K562 cells were harvested and dissociated into single‐cell suspensions. The cells were adjusted to the target cell density (4 × 10^4^ cells/mL) by dilution with RPMI‐1640 medium (pretreated with different concentrations of Mei) and mixed with 0.7% agar at a 1:1 ratio, respectively, to obtain a final concentration of 0.35% agar containing cells and the indicated concentrations of Mei (0, 2.5, 5, or 10 µM). For each well, 1 mL of 0.35% agar containing cells was added to 0.6% bottom agar in 6‐well plates and stored at 25 °C for 15 min to solidify the cell agar. After solidification, 0.5 mL per well of complete RPMI‐1640 medium was added to the cell agar, and the cells were cultured at 37 °C in 5% CO_2_. The medium was changed every 4 days. The colony size of each clone > 50 cells was observed under a microscope, and the colonies were photographed under a microscope. Assays were performed in triplicate.

### EdU Staining Assay

The growth of K562 cells incubated with 0, 2.5, 5, and 10 µM Mei for 24 h and PKMYT1‐knockdown cells were evaluated using the EdU Proliferation Kit (APEXBIO, USA), and the cells were stained and photographed by fluorescence microscopy (Echo, USA) according to the reagent manufacturer′s instructions and quantified using ImageJ, or the fluorescence intensity of the cells was measured by flow cytometry and quantified using FlowJo V10.6.2.

### JC‐1 Staining Assay

The mitochondrial membrane potential of K562 cells incubated with 0, 2.5, 5, and 10 µM Mei for 24 h and PKMYT1‐knockdown cells was evaluated using the JC‐1 kit (Biyotime, Shanghai), and the cells were stained and photographed by fluorescence microscopy (Echo, USA) according to the manufacturer′s instructions, and quantified using ImageJ.

### ROS Content Testing

The reactive oxygen contents of K562 cells incubated with 0 and 10 µM Mei for 24 h and PKMYT1‐knockdown K562 cells were evaluated using a ROS kit (Yeasen, China), and the cells were stained and photographed by fluorescence microscopy according to the reagent manufacturer′s instructions, and quantified using ImageJ.

### Flow Cytometry

For in vivo studies, bone marrow and blood samples were collected, lysed with erythrocyte lysis buffer (Solarbio, Beijing) on ice for 10 min, 1 mL of PBS (Solarbio, Beijing) was added to terminate the reaction, 450 g centrifugation was discarded for 5 min, repeated 2 times, cells were resuspended in 100 µL PBS, 2 µL of Human TruStain FcX blocking agent was added, blocked on ice for 10 min, and stained with anti‐human CD45 and anti‐human CD34 antibodies (Biolegend, San Diego, CA, USA) for 20 min. After staining, 1 mL of PBS was added to stop the reaction, 450 g centrifugation discarded the supernatant, repeated twice, and cells were resuspended in 400 µL PBS. The cells were detected using a Cytek Guava easyCyte HT instrument (Luminex, USA) and analyzed by FlowJo software (version 10.6.2).

For cell cycle analysis, wild‐type and PKMYT1‐knockdown K562 cells were fixed with ethanol and stained with propidium iodide. The cells were detected using a Cytek Guava easyCyte HT instrument (Luminex, USA) and analyzed by ModfitLT 5 software.

### Oxygen Consumption Rate Measurements

The oxygen consumption rate (OCR) was measured using a Seahorse XF24 analyzer (Angilent, California, USA). K562 cells were suspended in XF RPMI medium supplemented with 10 mM glucose, 1 mM pyruvate, and 2 mM glutamine. A total of 100 000 cells were seeded per well of a Seahorse XF24 cell culture plate (100 µL volume) precoated with Cell‐Tak (Corning, USA). The cells were allowed to adhere for a minimum of 30 min in a CO_2_‐free incubator at 37 °C, after which 400 µL of XF RPMI medium (Angilent, USA) was added to each well. The plate was allowed to equilibrate for 10 min in the incubator without additional CO_2_ supplementation before being transferred to the Seahorse XF24 analyzer. The OCR was measured at baseline and following sequential injections of i) oligomycin (1.5 µM), an ATP synthase inhibitor; ii) carbonyl cyanide‐4‐(trifluoromethoxy)phenylhydrazone (FCCP) (1.0 µM), a mitochondrial uncoupler; and iii) antimycin A and rotenone (0.5 µM; all Angilent), complex III and complex I inhibitors, respectively. This enabled to measure the OCR coupled to ATP production. The OCRs were normalized to the number of cells.

### Western Blotting

Cell lysates were obtained by incubating the cells with 0.2% NP40/PBS for 20 min on ice, and then frozen in liquid nitrogen 3 times. After centrifugation at 15 000 rpm for 20 min at 4 °C, the supernatant was collected. The protein concentration of the supernatant was quantified using a Pierce BCA protein assay kit. A fixed volume of cell lysate was boiled with SDS‐PAGE loading buffer for 10 min at 95 °C, separated by 8–12% SDS‐PAGE, transferred to polyvinylidene difluoride (PVDF) membranes (Millipore), incubated with skim milk, and incubated with primary antibodies specific for target proteins and horseradish peroxidase (HRP)‐conjugated secondary antibodies (Proteintech). Super ECL Plus (Applygen) was used to detect the intensity of the proteins. The antibodies used are listed in Table S2 (Supporting Information).

### Animal Studies

For the K562 ectopic xenograft tumor model, BALB/c nude mice (4–5 weeks old, female) were purchased from Charles River (Beijing, China). After 1 week of adaptation, the mice were randomly divided into three groups. About 100 µL of wild‐type or PKMYT1‐knockdown K562 cells (3 × 10^6^ cells) were injected into the right flanks of the mice to generate xenograft tumors. After subcutaneous inoculation of cells for 7–10 days, when the wild‐type tumors were ≈100 mm^3^, the mice were treated with vehicle or Mei. The treatments were as follows: control group (vehicle control, i.g., once daily, n = 12), SG group (vehicle control, i.g., once daily, n = 12), and SG‐Mei group (treated with Mei 150 mg kg^−1^, i.g., once daily, n = 12). The body weights of the nude mice were recorded every two days. The two perpendicular diameters of the tumors were measured and the tumor volume was calculated by using the formula V = 0.5 × length (mm) × width (mm).^2^ Tumor weights were recorded after 14 days of Mei treatment when the mice were sacrificed. Mice were anesthetized and euthanized when the average tumor diameter was near 20 mm, and tumor volume approached 1500 mm^3^ but did not exceed 2000 mm^3^. All animal care and experiments were approved by the Animal Care & Welfare Committee Institute of Materia Medica, CAMS & PUMC (approval number: 00008498).

For the K562 tumor xenograft leukemia model, NOD‐SCID mice (6‐8 weeks old, female) were purchased from Beijing HFK Biosciences Co., Ltd. (Beijing, China). The mice were maintained under specific pathogen‐free (SPF) conditions in the animal facility of the Institute of Materia. After receiving 1 Gy of radiation, NOD‐SCID mice were injected i.v. with 1×10^7^ K562 wild‐type and PKMYT1‐knockdown cells. The survival rate of these mice was monitored for 70 days. Mice were euthanized if they lost more than 20% of their body weight or were near death. In another xenograft leukemia model, the mice were anesthetized at the end of the experiment, and blood and bone marrow were collected for analysis. Bone marrow and blood cell smears were performed and stained with a modified Giemsa stain (Beyotime, Shanghai). All animal care and experiments in this model were approved by the Animal Care & Welfare Committee Institute of Materia Medica, CAMS & PUMC (approval numbers: 00002455, 00002500).

### Bioinformatics Analysis and Statistical Analysis

All data sets used in this study are publicly available. The expression of mRNAs in the Gene Expression Omnibus (GEO) database was obtained from the GEO website. The PPI network was constructed using the Search Tool for the Retrieval of Interacting Genes (STRING) database.

All the statistical analyses and data fitting were performed using GraphPad Prism software 8.0. Statistical significance was assessed via Student's t‐test. P values <0.05, <0.01, or <0.001 were considered to indicate statistical significance. Further details of each statistical analysis are provided in the figure legends.

## Conflict of Interest

The authors declare no conflict of interest.

## Author Contributions

S.S.Y., Y.L., J.J., and Y.B.L. designed and directed this study and revised the manuscript. Z.X.Z., F.F.L., and S.Y.L. conducted experiments, performed data analysis, and wrote the manuscript. Q.Y.S., C.Y.T., X.J.W., and M.L. assisted in experiments. All authors contributed to the discussion and interpretation of the results.

## Supporting information



Supporting Information

## Data Availability

The data that support the findings of this study are available from the corresponding author upon reasonable request.
