# Supplementary material for: Meisoindigo Acts as a Molecular Glue to Target PKMYT1 for Degradation in Chronic Myeloid Leukemia Therapy
Source: Adv Sci (Weinh). 2025 Apr 25;12(21):2413676. doi: 10.1002/advs.202413676 (PMC12140364; doi:10.1002/advs.202413676)
Supplement: Supplementary file 1 — Supporting Information [file ADVS-12-2413676-s001.docx]

**Supporting Information**

**Meisoindigo Acts as a Molecular Glue to Target PKMYT1 for Degradation in Chronic Myeloid Leukemia Therapy**

*Zhao-Xin Zhang^1,2^, Shu-Ying Li^1,2^, Fang-Fei Li^1,2^, Qin-Yan Shi^1^, Cheng-yong Tan^1^, Xiao-Jing Wang^1^, Mi Li^1^, Yun-Bao Liu^1*^, Jing Jin^1*^, Yong Li^1*^ & Shi-Shan Yu^1*^*

^1^State Key Laboratory of Bioactive Substance and Function of Natural Medicines, Institute of Materia Medica, Chinese Academy of Medical Sciences & Peking Union Medical College, Beijing 100050, China.

^2^These authors contributed equally to this work.

*e-mail: [yushishan@imm.ac.cn](mailto:yushishan@imm.ac.cn) (Shi-Shan Yu), [liyong@imm.ac.cn](mailto:liyong@imm.ac.cn) (Yong Li), [rebeccagold@imm.ac.cn](mailto:rebeccagold@imm.ac.cn) (Jing Jin), and [liuyunbao@imm.ac.cn](mailto:liuyunbao@imm.ac.cn) (Yun-Bao Liu).

Contents

[1. General information 1](#_Toc187095936)

[2. Chemical synthesis of compounds 1](#_Toc187095937)

[2.1. Synthesis of meisoindigo-alkyne probe (MP) 1](#_Toc187095938)

[2.2. Synthesis of Mei-BME 2](#_Toc187095939)

[3. Supplementary figures 4](#_Toc187095940)

[4. Supplementary tables 13](#_Toc187095941)

[Table S1. Primers used for real-time quantitative PCR assays 13](#_Toc187095942)

[Table S2. Antibodies for immunoblotting 13](#_Toc187095943)

[Table S3. Antibodies for flow cytometry 14](#_Toc187095944)

[5. Appendix 14](#_Toc187095945)

[5.1. NMR Spectra 15](#_Toc187095946)

[5.2. High resolution mass spectra 17](#_Toc187095947)

**1. General information**

The click chemistry tris[(1-benzyl-1H-1,2,3-triazol-4-yl)methyl]amine (TBTA), Tris(2-carboxyethyl)phosphine (TCEP) were purchased from Sigma-Aldrich (St. Louis, MO, USA). CuSO_4_ was purchased from Acros Organics™. TAMRA Azide and Biotin-PEG3-Azide were purchased from Click Chemistry Tools (Scottsdale, USA). Pierce^TM^ Streptavidin Magnetic Beads, High Capacity Streptavidin Agarose Resin, TMT 10plex^TM^ label Reagent Set (90110), and Pierce^TM^ C18 Tips, were purchased from Thermo Scientific. High Affinity Ni-NTA Resin was purchased from GenScript (Nanjing, China). Centrifugal filters and Transfer Membrane were purchased from Merck Millipore Ltd. (Tullagreen, Carrigtwohill, Co Cork IREL and Rev). Flag-Nanoab-Agarose was purchased from LABLEAD (Beijing, China). PKMYT1 human recombinant protein (#TP322657M) and TRIM25 human recombinant protein (#TP303757) were purchased from Ori Gene. ^1^H NMR and ^13^C NMR spectra were measured on a Bruker AVANCEIII 400 MHz, Bruker AVANCEIII 700 MHz, and Q One 400 MHz spectrometer. Chemical shifts were given in ppm, and coupling constants were given in Hertz. Western blots were captured with Tanon 5200 Multi (Tanon, Shanghai) or MiniChemiTM610 (Sinsage, Beijing) chemiluminescence image analysis system. HRESIMS data were obtained with an Agilent 6500 Series Q-TOF or a Thermo-Scientific Q Exactive Focus LC/MS spectrometer.

**2. Chemical synthesis of compounds**

**2.1. Synthesis of meisoindigo-alkyne probe (MP)**

**Scheme S1.** Synthesis of MP.

Isoindigo (50 mg，191 μmol) and K_2_CO_3_ (52.7 mg，382 μmol) were dissolved in DMF (3.5 mL), followed by addition of 6-iodo-1-hexyne (43.7 mg，210 μmol). The solution was stirred at room temperature for 4h. The reaction mixture was diluted with H_2_O (60 mL) and extracted with EtOAc (3×60 mL). The combined EtOAc extracts were dried with Na_2_SO_4_, concentrated and the residue obtained was purified by preparative TLC (PE-EtOAc, 5:1) to afford 29.8 mg (46% combined yield).

^1^H-NMR (400 MHz，DMSO-d6) δ 10.90 (s, 1H, -NH-), 9.08 (m, 2H), 7.37 (m, 2H), 7.11-6.93 (m, 3H), 6.84 (m, 1H), 3.78 (brs, 2H), 2.75 (s, 1H), 2.22 (brs, 2H), 1.71 (brs, 2H), 1.50 (brs, 2H); ^13^C-NMR (100 MHz，DMSO-d6) δ 168.84, 167.14, 144.26, 144.20, 133.93, 132.88, 132.55, 132.11, 129.48, 129.13, 121.68, 121.64, 121.17, 120.88, 109.60, 108.54, 84.18, 71.46, 38.80, 26.09, 25.33, 17.33; HR-ESI-MS *m*/*z* 343.14352 [M+H] ^+^, (calcd for C_22_H_19_O_2_N_2_, 343.14410).

**2.2. Synthesis of Mei-BME**

**Scheme S2.** Synthesis of Mei-BME.

Mei (40 mg，145 μmol) was dissolved in DMSO (0.9 mL) and PBS (0.3 mL). Followed by addition of β-mercaptoethanol (BME, 120 µL, 10.6 equiv). The solution was stirred at room temperature for 30 min. The red of meisoindigo disappeared, complete conversion to the adducts was observed. The reaction mixture was diluted with H_2_O (20 mL) and extracted with EtOAc (3×20 mL). The combined EtOAc extracts were dried with Na_2_SO_4_, concentrated and the residue obtained was purified by preparative TLC (toluene: 2-propanol, 8:1) to afford 31.5 mg (61% combined yield). The product was a mixture with different stereo configurations.

^1^H NMR (400 MHz, DMSO-d6) δ 10.61 (s, 0.55H, -NH-), 10.20 (s, 0.45H, -NH-), 7.85 (t, *J* = 8.1 Hz, 1H), 7.41 (t, *J* = 7.7 Hz, 0.53H), 7.31 (t, *J* = 7.7 Hz, 0.59H), 7.24 (td, *J* = 7.7, 1.2 Hz, 0.49H), 7.17-7.11 (m, 1.15H), 7.08 (t, *J* = 7.6 Hz, 0.55H), 6.99 (t, *J* = 8.1 Hz, 1.13H), 6.81 (d, *J* = 4 Hz, 0.57H), 6.79 (d, *J* = 4 Hz, 0.48H), 6.73 (t, *J* = 7.6 Hz, 0.53H), 6.63 (t, *J* = 7.6 Hz, 0.66H), 6.10 (d, *J* = 7.6 Hz, 0.48H), 6.02 (d, *J* = 7.6 Hz, 0.53H), 4.90 (t, *J* = 5.4 Hz, 1.10H), 4.86 (t, *J* = 5.4 Hz, 0.90H), 4.21 (s, 0.55H), 4.19 (s, 0.45H), 3.64-3.57 (m, 2H), 3.17 (s, 1.53H), 2.82 (s, 1.74H), 2.79 (t, *J* = 6.6 Hz, 2H); ^13^C-NMR (175 MHz，DMSO-d6) δ 175.70, 174.45, 174.20, 172.51, 144.78, 143.41, 143.31, 141.93, 129.51, 129.38, 128.94, 128.87, 127.04, 127.02, 126.65, 126.38, 125.25, 124.52, 122.89, 122.78, 121.88, 121.79, 121.16, 121.14, 109.78, 109.35, 108.69, 108.35, 59.78, 59.61, 53.13, 52.61, 47.95, 47.13, 31.02, 30.96, 26.33, 25.81; HR-ESI-MS *m*/*z* 355.1106 [M+H] ^+^, (calcd for C_19_H_18_N_2_O_3_S, 355.1111).

**3. Supplementary figures**


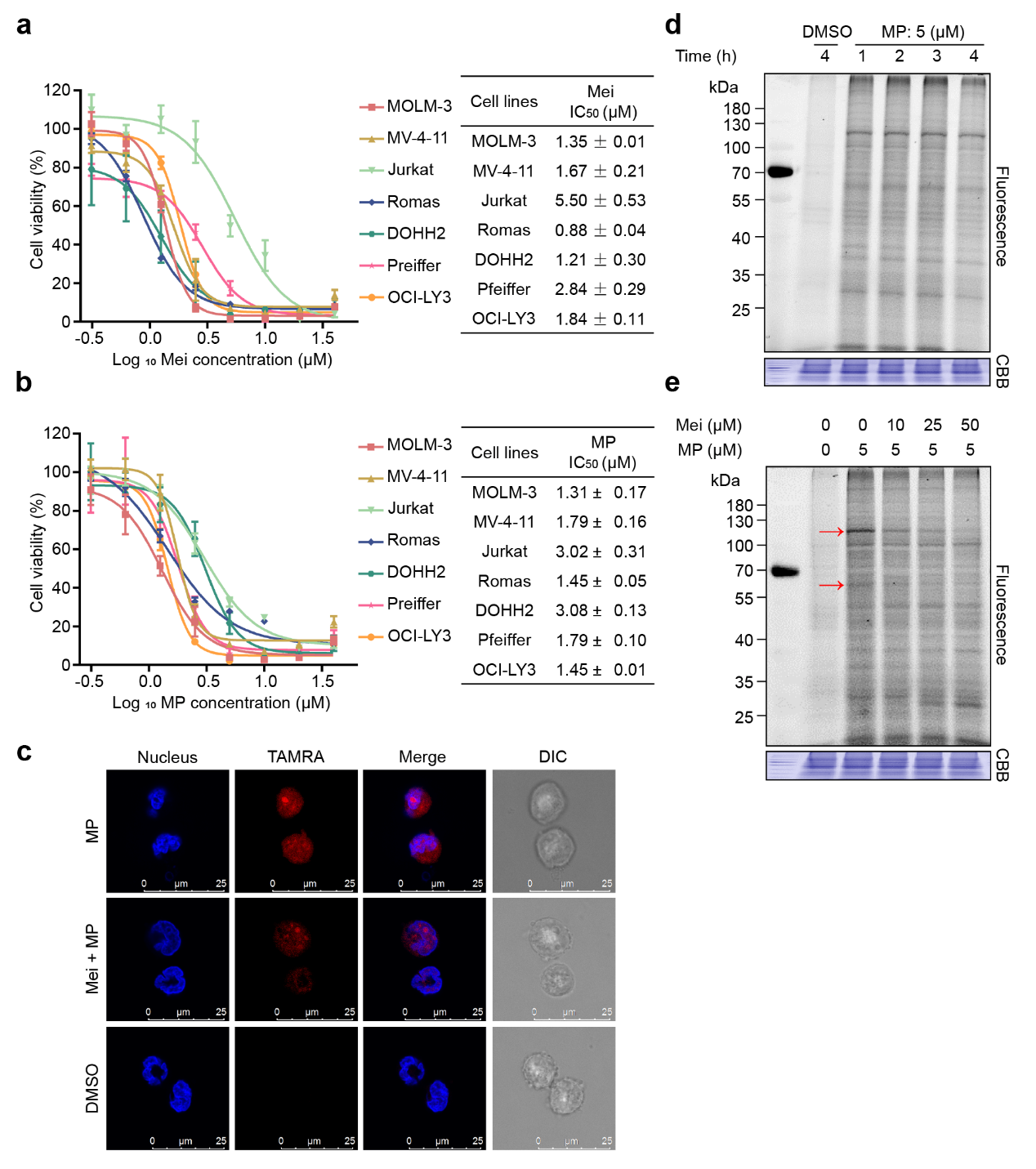


**Figure S1.** MP can be used in activity-based proteomic analysis assay. a) Cell viability of different tumor cells upon incubation with Mei for 72 h (n = 3). b) Cell viability of different tumor cells upon incubation with MP for 72 h (n = 3). c) Confocal microscopy imaging of K562 cells treated with MP (10 μM) together with or without Mei (50 μM), with DMSO used as the control. d) *In situ* time-dependent fluorescence labeling of MP in K562 cells. e) *In situ* competitive fluorescence labeling of MP with or without Mei pretreatment in K562 cells. The data are presented as the means ± SDs.


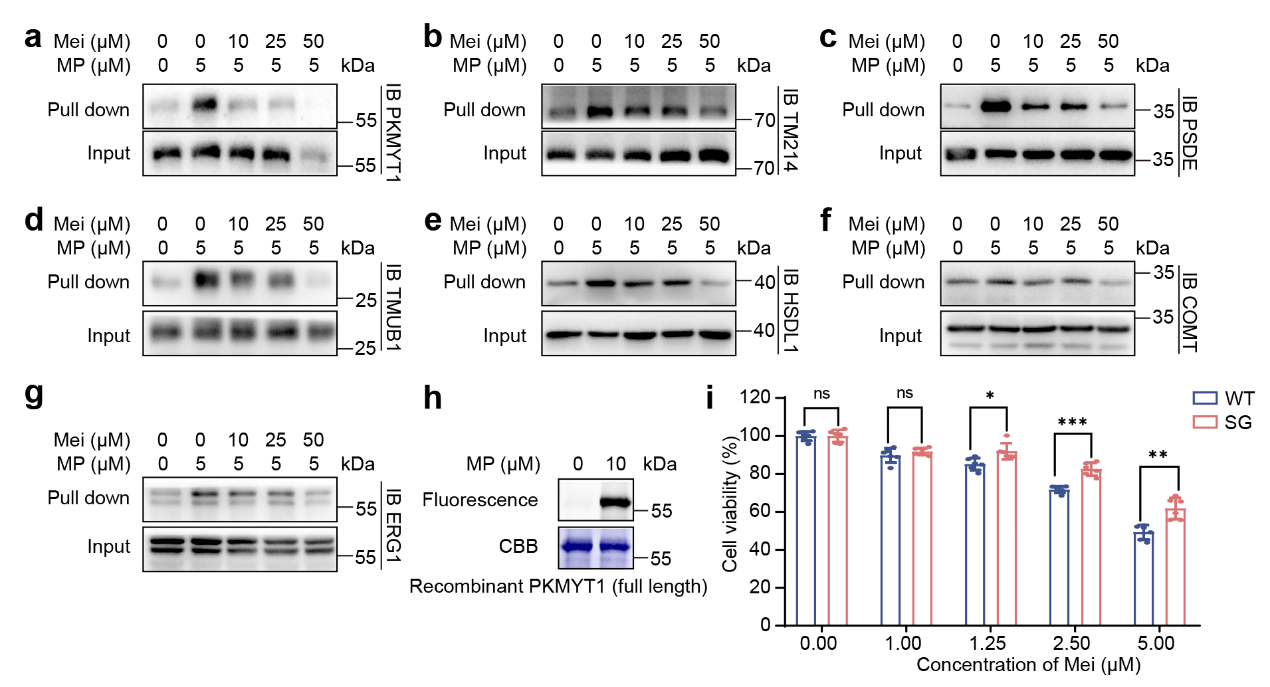


**Figure S2.** PKMYT1 is the target of Mei. a-g) Western blot analysis of the seven potential target proteins in protein affinity pull-down assay in K562 cells, and the cells were treated with MP (5 μM) with or without Mei (10, 25 or 50 μM). h) Fluorescence labeling of full-length recombinant PKMYT1 with MP (10 μM). i) Viability of wild-type and PKMYT1-knockdown K562 cells after 72 h treatment with Mei at various concentrations (n = 6). The data are presented as the means ± SDs. Statistical significance was assessed via two-tailed unpaired Student's t-test. NS, not significant; *P < 0.05, **P < 0.01, ***P < 0.001 vs. the control group.


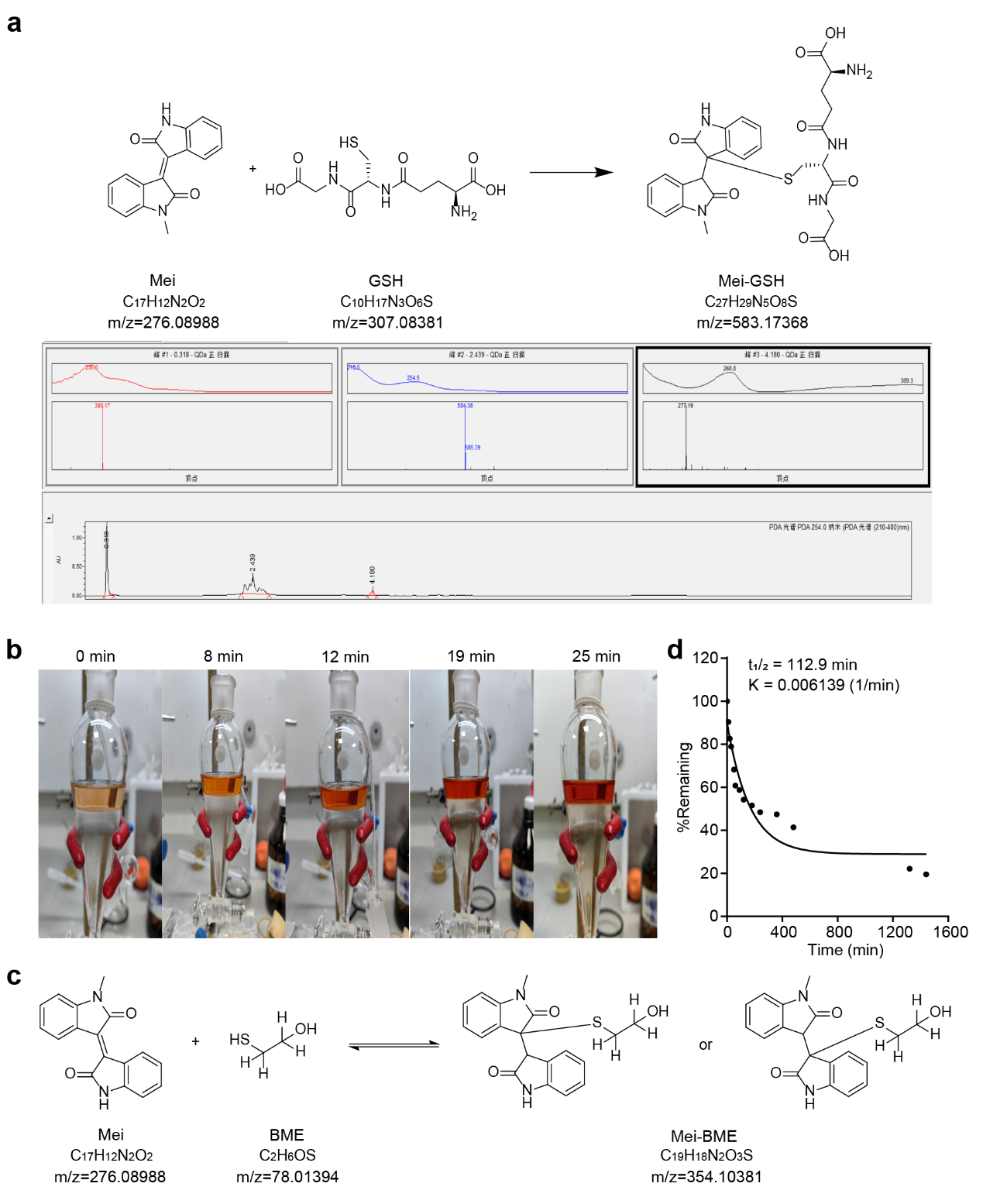


**Figure S3.** The interaction between Mei and the sulfhydryl group is reversibly covalent. a) The MS analysis of the reaction of Mei and GSH. Glutathione (GSH, 1 mM) was incubated with Mei (1 mM) for 1 h at room temperature. b) Color change of the organic phase when the reaction mixture of Mei and cysteine was extracted. c) The synthesis process of Mei-BME. d) The decomposition curve of Mei-BME complex in PBS.


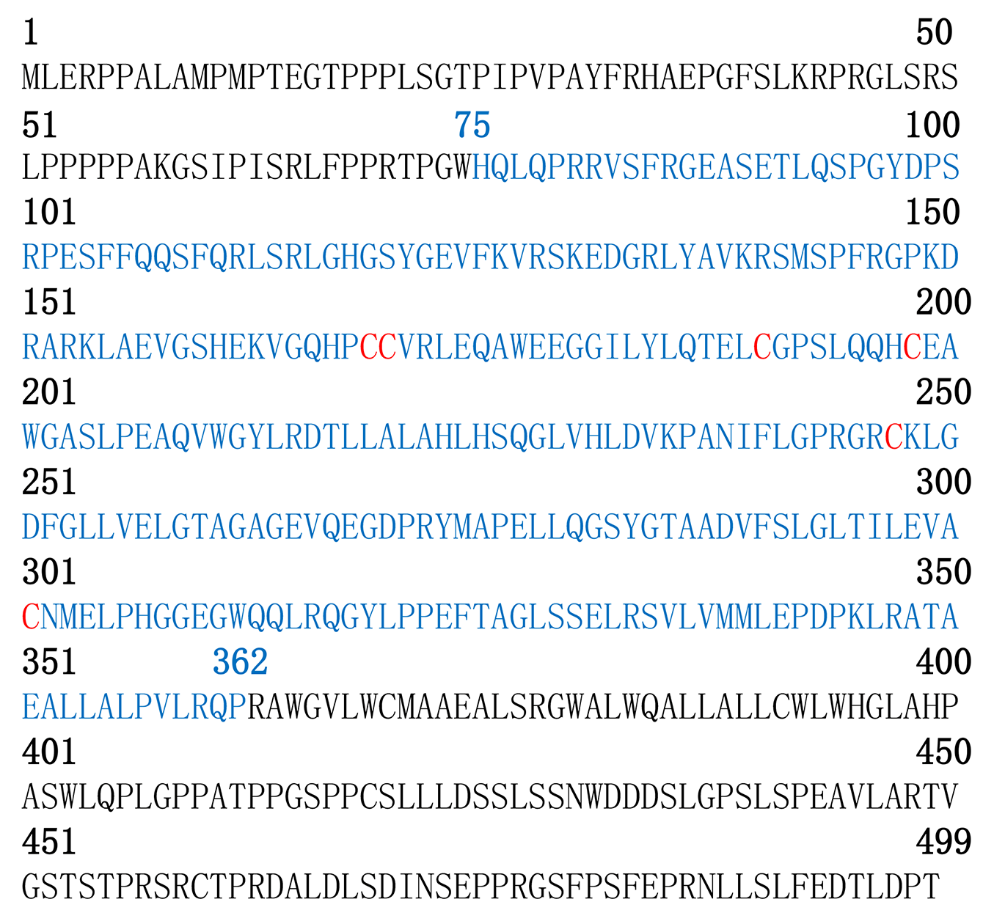


**Figure S4.** Amino acid sequence of the human PKMYT1 protein. The PKMYT1 (75-362) sequence is shown in blue and the PKMYT1 (75-362) cysteine residues are shown in red.


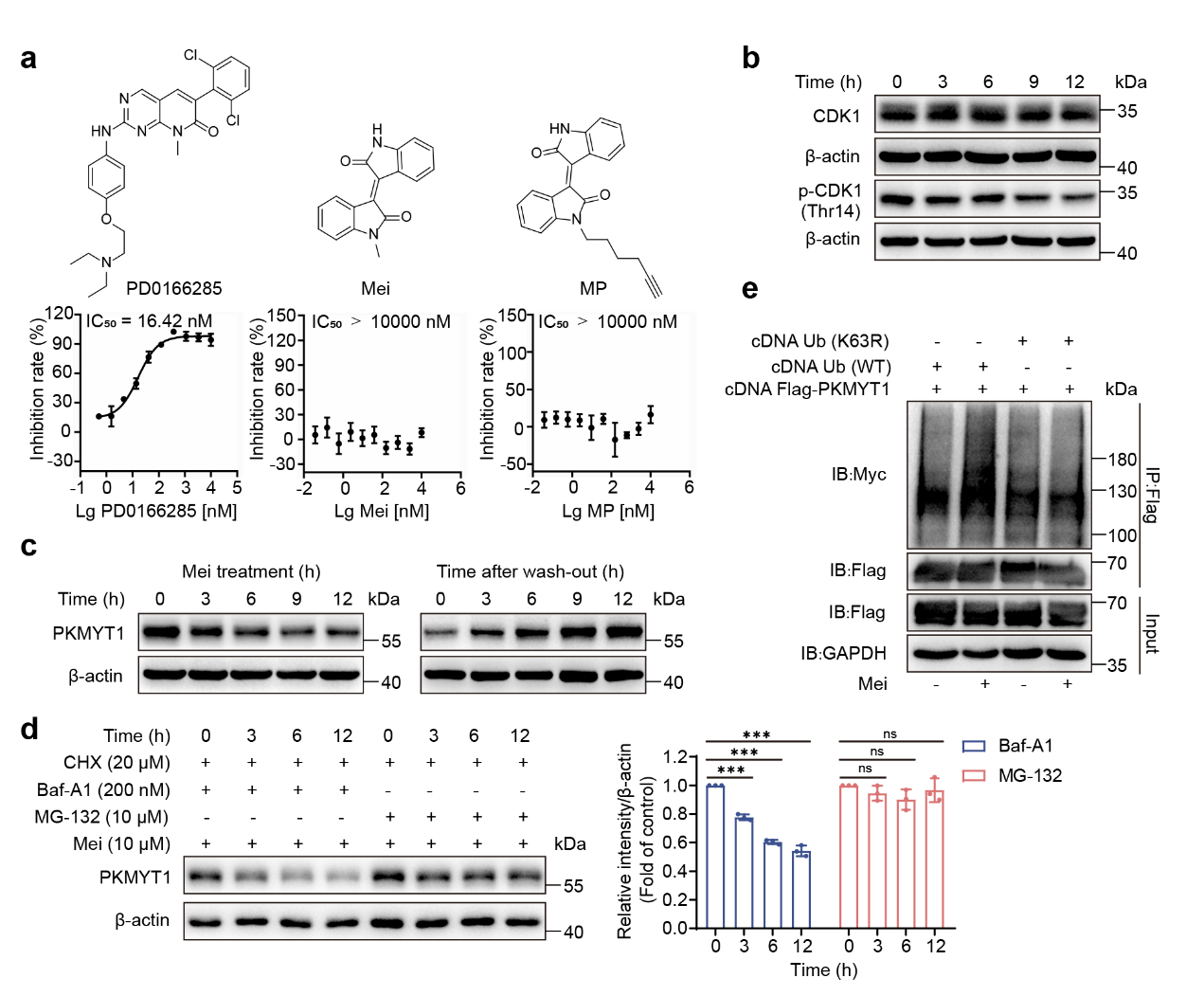


**Figure S5.** Mei promotes K48-linked ubiquitination leading to PKMYT1 degradation. a) Mei and MP did not inhibit the enzymatic activity of PKMYT1 compared with the positive control drug PD0166285 (IC_50_ = 16.42 nM). b) Western blot analysis of p-CDK1 (Thr 14) and CDK1 protein expression in K562 cells treated with Mei (10 μM) at different time points. c) K562 cells were treated with Mei (10 μM) for the indicated times. After 12 h of exposure, the cells were washed and the PKMYT1 protein levels were evaluated by Western blot analysis. d) Western blot analysis of PKMYT1 protein expression in K562 cells treated with CHX (20 μM), Mei (10 μM), MG-132 (10 μM) or Baf-A1 (200 nM) at different time points (n = 3). e) Co-IP assays revealed that Mei did not induce K63-type ubiquitination of PKMYT1 in cells. The data are presented as the means ± SDs. Statistical significance was assessed via two-tailed unpaired Student's t-test. NS, not significant; ***P < 0.001 vs. the control group.


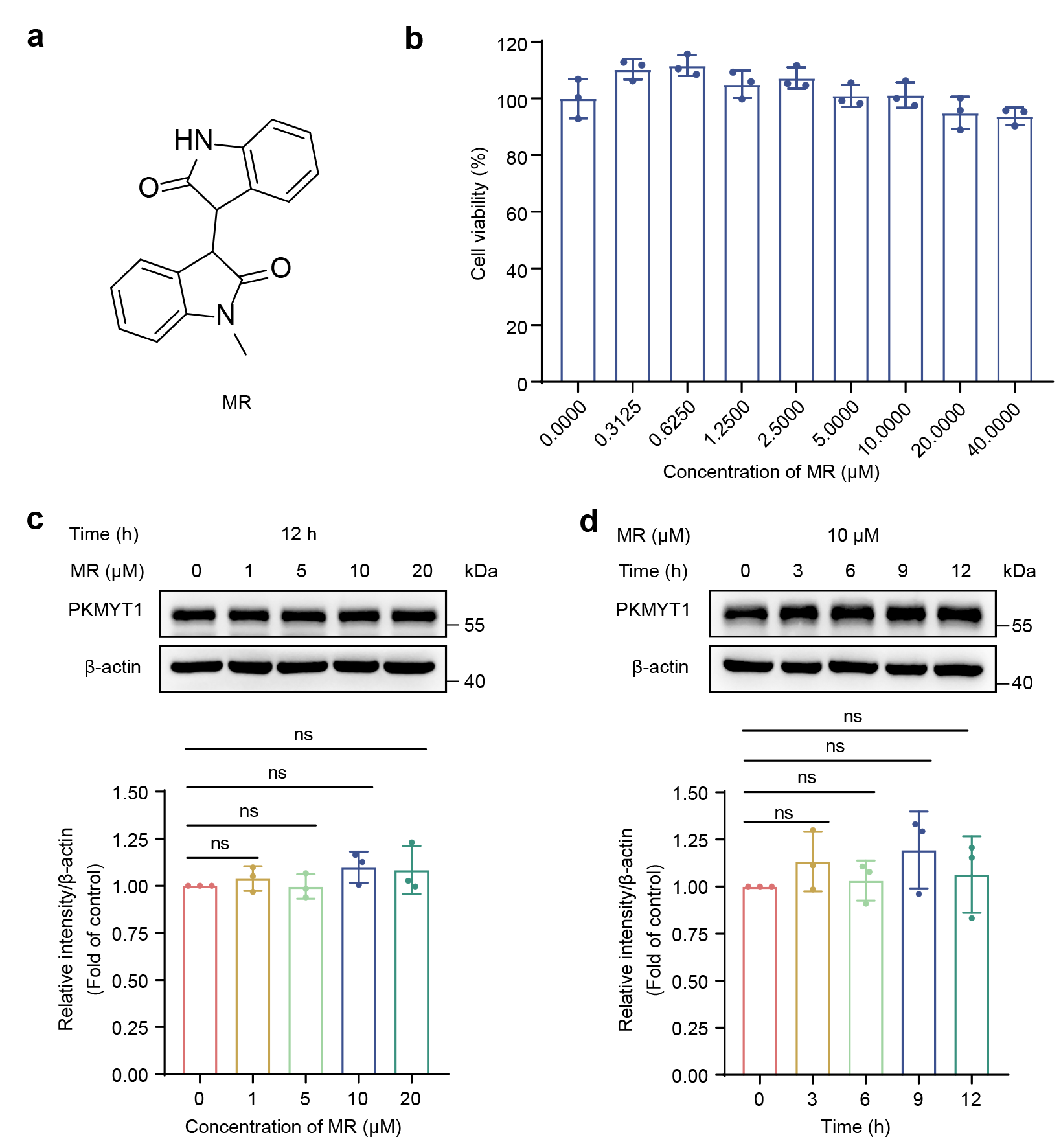


**Figure S6.** The covalent bond formation between Mei and PKMYT1 is necessary for PKMYT1 degradation. a) Chemical structure of the reduction product of Mei (MR). b) Cell viability of K562 cells treated with MR for 72 h (n = 3). c) Western blot analysis of PKMYT1 protein levels in K562 cells treated with different concentrations of MR for 12 h (n = 3). d) Western blot analysis of PKMYT1 protein levels in K562 cells treated with MR (10 μM) at different time points (n = 3). The data are presented as the means ± SDs. Statistical significance was assessed via two-tailed unpaired Student's t-test, P > 0.05, not significant (NS) vs. the control group.


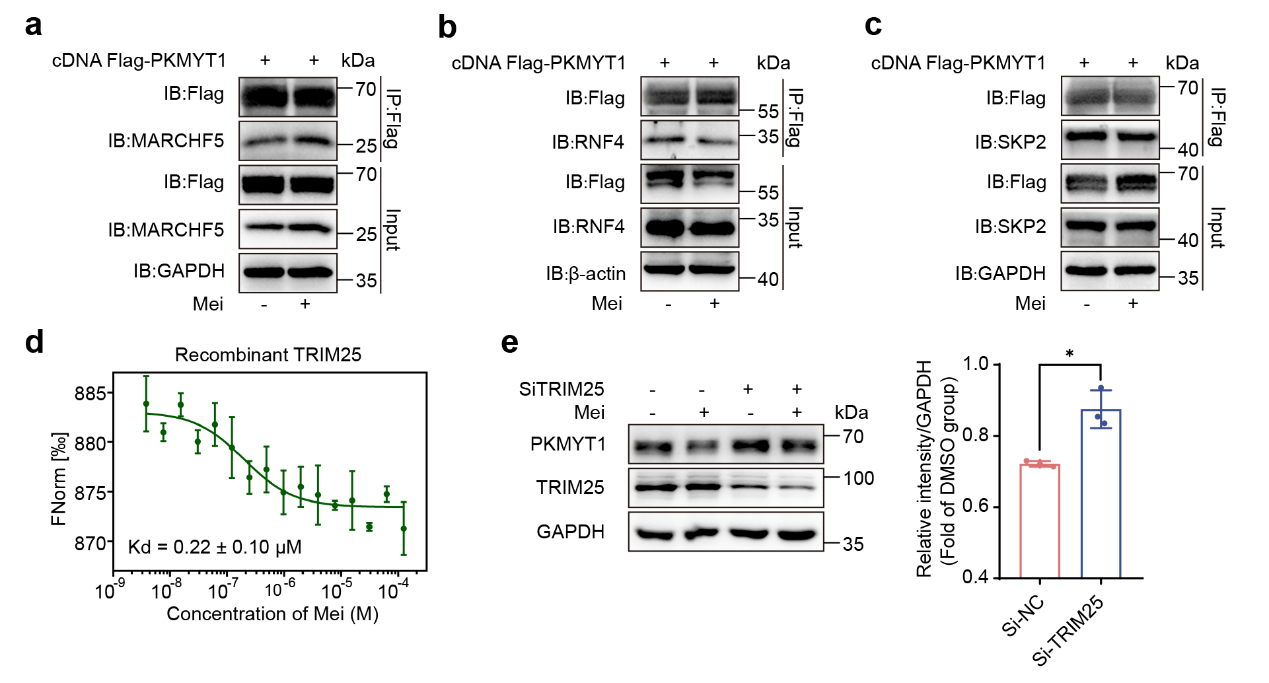


**Figure S7.** Mei could enhance the PKMYT1-TRIM25 interaction and TRIM25 involved in PKMYT1 degradation. a-c) Co-IP assays reveal interactions between PKMYT1 and E3 ligases MARCHF5, RNF4, or SKP2 in HEK293T cells. HEK293T cells overexpressing Flag-PKMYT1 were treated with Mei (10 μM) or DMSO for 3 h. d) MST assay of Mei binding to recombinant TRIM25 (n = 3). e) Mei-induced PKMYT1 degradation was partially reversed by TRIM25 knockdown.


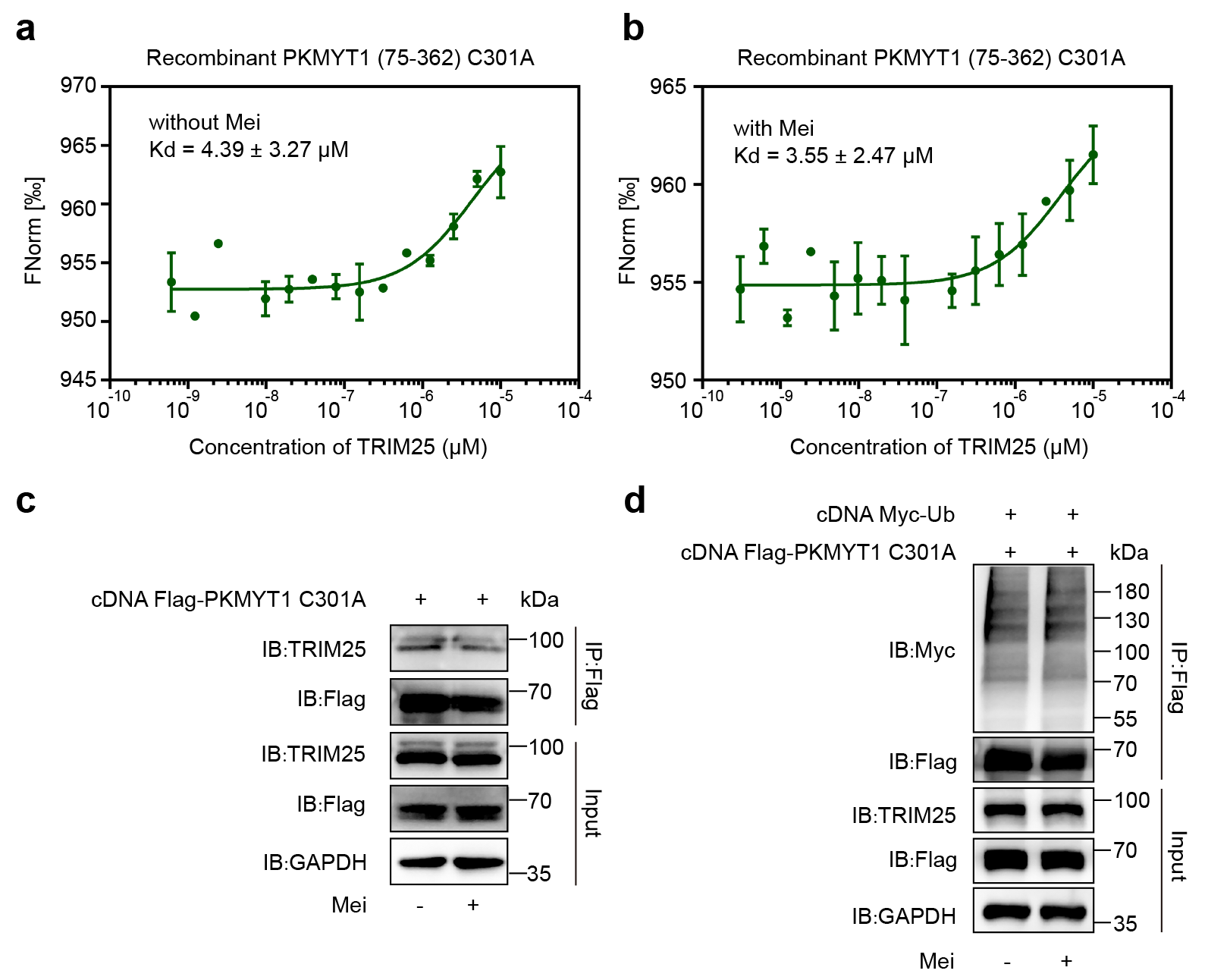


**Figure S8.** Mei could not enhance the PKMYT1 (C301A)-TRIM25 interaction nor promote PKMYT1 (C301A) ubiquitination. a) MST assay of TRIM25 binding to the recombinant PKMYT1 (75-362) C301A mutant protein (n = 3). b) MST assay of TRIM25 binding to the recombinant PKMYT1 (75-362) C301A mutant protein preincubated with Mei (n = 3). c) Co-IP assay reveals the interaction between PKMYT1 (C301A) and E3 ligases TRIM25 in HEK293T cells. HEK293T cells overexpressing Flag-PKMYT1 (C301A) were treated with Mei (10 μM) or DMSO for 3 h. d) Mei could not promote PKMYT1 (C301A) ubiquitination. HEK293T cells were cotransfected with the indicated plasmids for 24 h and then treated with DMSO or Mei (10 μM) for 3 h.


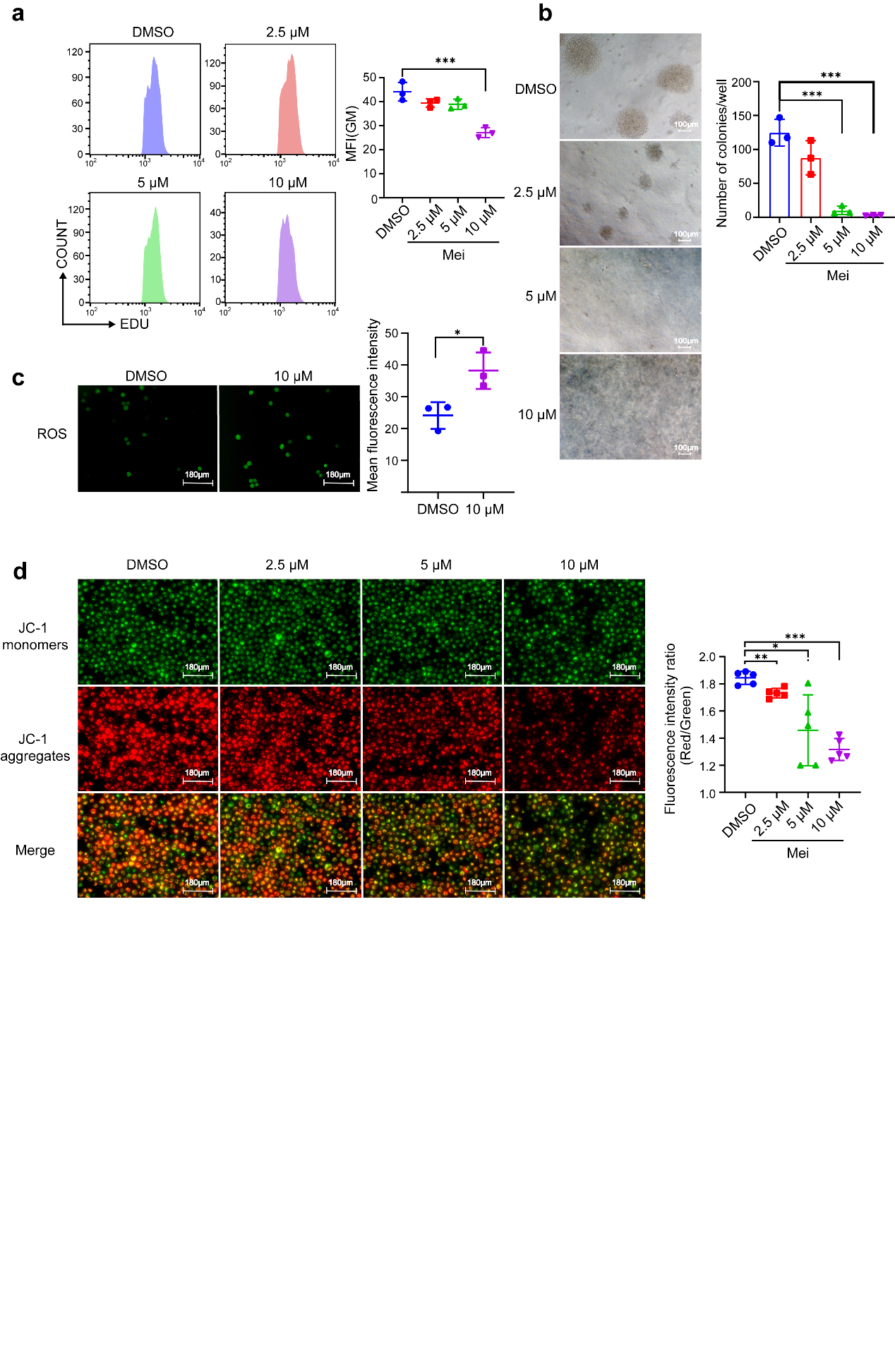


**Figure S9.** Mei treatment promotes the early apoptosis of K562 cells and inhibits their proliferation. a) EdU assay for cell proliferation of wild-type K562 cells treated with 0, 2.5, 5 and 10 μM Mei for 24 h (n = 3). b) Number of clones of wild-type K562 cells treated with 0, 2.5, 5, and 10 μM Mei (n = 3). c) ROS levels in wild-type K562 cells treated with 0 and 10 μM Mei for 24 h (n = 3). d) Levels of the mitochondrial membrane potential in wild-type K562 cells treated with 0, 2.5, 5, and 10 μM Mei for 24 h (n = 5). The data are presented as the means ± SDs. Statistical significance was assessed via two-tailed unpaired Student's t-test. *P < 0.05, **P < 0.01, ***P < 0.001 vs. the control group.


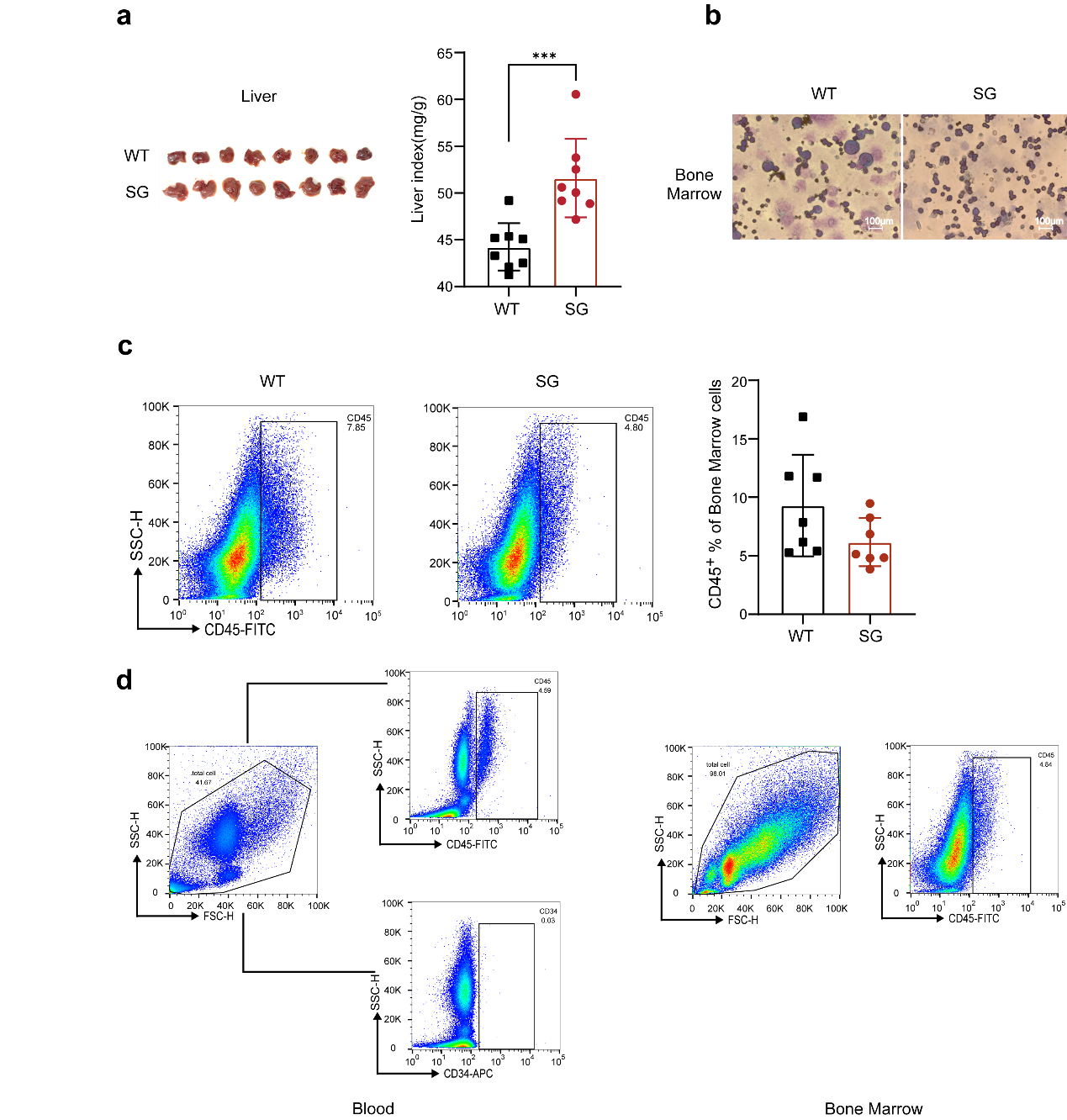


**Figure S10.** PKMYT1 knockdown slows the leukemogenesis and malignant proliferation of cancer cells. a) Liver indices of mice in the wild-type and PKMYT1-knockdown CML groups in the orthotopic xenograft model (n = 8). b) Bone marrow smears of mice in the wild-type and PKMYT1-knockdown groups (n = 8). c) Human CD45^+^ cell content in the bone marrow of PKMYT1-knockdown and wild-type group mice (n = 7). d) Peripheral blood and bone marrow cell flow cytometry gating strategy. The data are presented as the means ± SDs. Statistical significance was assessed via two-tailed unpaired Student's t-test, ***P < 0.001 vs. the wild-type group.

**4. Supplementary tables**

**Table S1. Primers used for real-time quantitative PCR assays**

| Primer name | Primer sequence (5′–3′) |
| --- | --- |
| PKMYT1-Forward | CATGGCTCCTACGGAGAGGT |
| PKMYT1-Reverse | ACATGGAACGCTTTACCGCAT |
| GAPDH-Forward | GGGTGTGAACCATGAGAAGT |
| GAPDH-Reverse | GGCATGGACTGTGGTCATGA |

**Table S2. Antibodies for immunoblotting**

| Antibody | Vendor | Catalogue number | Dilution |
| --- | --- | --- | --- |
| PKMYT1 | Proteintech | 67806-1-lg | 1:5000 |
| PSMD14 (PSDE) | Cell Signaling Technology | 4197S | 1:1000 |
| TMEM214 | Proteintech | 20125-1-AP | 1:1000 |
| HSDL1 | Proteintech | 16988-1-AP | 1:1000 |
| SQLE (ERG1) | Proteintech | 12544-1-AP | 1:1000 |
| TUMB1 | abcam | ab180586 | 1:10000 |
| COMT | Proteintech | 14754-1-AP | 1:1000 |
| CDK1 | Proteintech | 19532-1-AP | 1:4000 |
| p-CDK1 (Thr14) | Cell Signaling Technology | 2543S | 1:1000 |
| TRIM25 | Proteintech | 12573-1-AP | 1:1000 |
| MARCH5 (MARCHF5) | Cell Signaling Technology | 19168 | 1:1000 |
| SKP2 | Proteintech | 15010-1-AP | 1:2000 |
| RNF4 | Proteintech | 17810-1-AP | 1:1000 |
| CDT1 | Cell Signaling Technology | 8064S | 1:1000 |
| Thymidine Kinase 1 | Cell Signaling Technology | 28755S | 1:1000 |
| Cyclin A2 | Cell Signaling Technology | 91500S | 1:1000 |
| Cyclin B1 | Cell Signaling Technology | 12231T | 1:1000 |
| Geminin | Cell Signaling Technology | 52508T | 1:1000 |
| GAPDH | Proteintech | 60004-1-Ig | 1:50000 |
| β-actin | Immunoway | YM3028 | 1:40000 |
| Myc tag | Proteintech | 16286-1-AP | 1:5000 |
| DYKDDDDK tag | Proteintech | 66008-04-lg | 1:5000 |
| Goat Anti-Rabbit IgG (H+L) | Proteintech | SA00001-2 | 1:5000 |
| Goat Anti-Mouse IgG (H+L) | Proteintech | SA00001-1 | 1:5000 |

**Table S3. Antibodies for flow cytometry**

| Antibody | Vendor | Catalogue number |
| --- | --- | --- |
| FITC anti-human CD45 | Biolegend | 304006 |
| APC anti-human CD34 | Biolegend | 343510 |
| Human TruStain FcX | Biolegend | 422301 |

**5. Appendix**

**5.1. NMR Spectra**

**^1^H-NMR spectra of MP in DMSO-d6**

**^13^C-NMR spectra of MP in DMSO-d6**

**^1^H-NMR spectra of Mei-BME in DMSO-d6**

**^13^C-NMR spectra of Mei-BME in DMSO-d6**

**5.2. High resolution mass spectra**


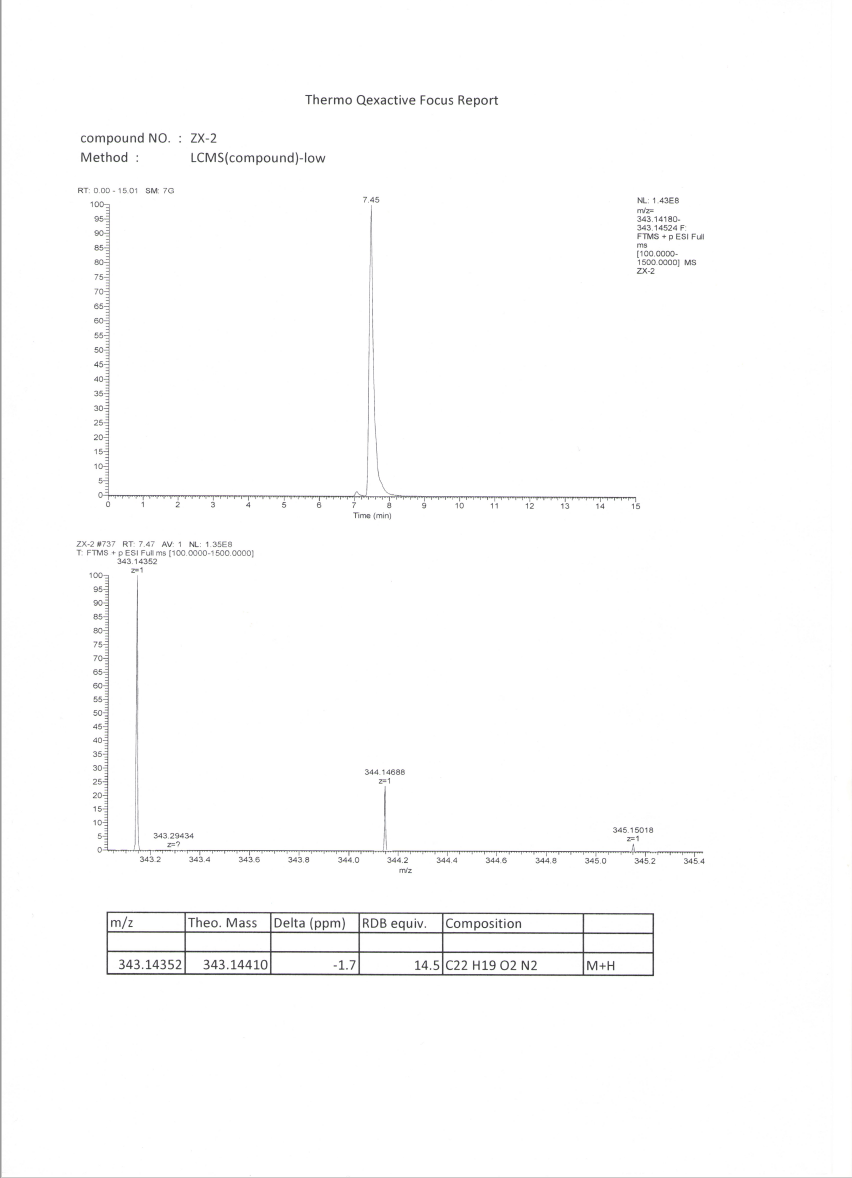


**HR-ESI-MS spectra of MP**


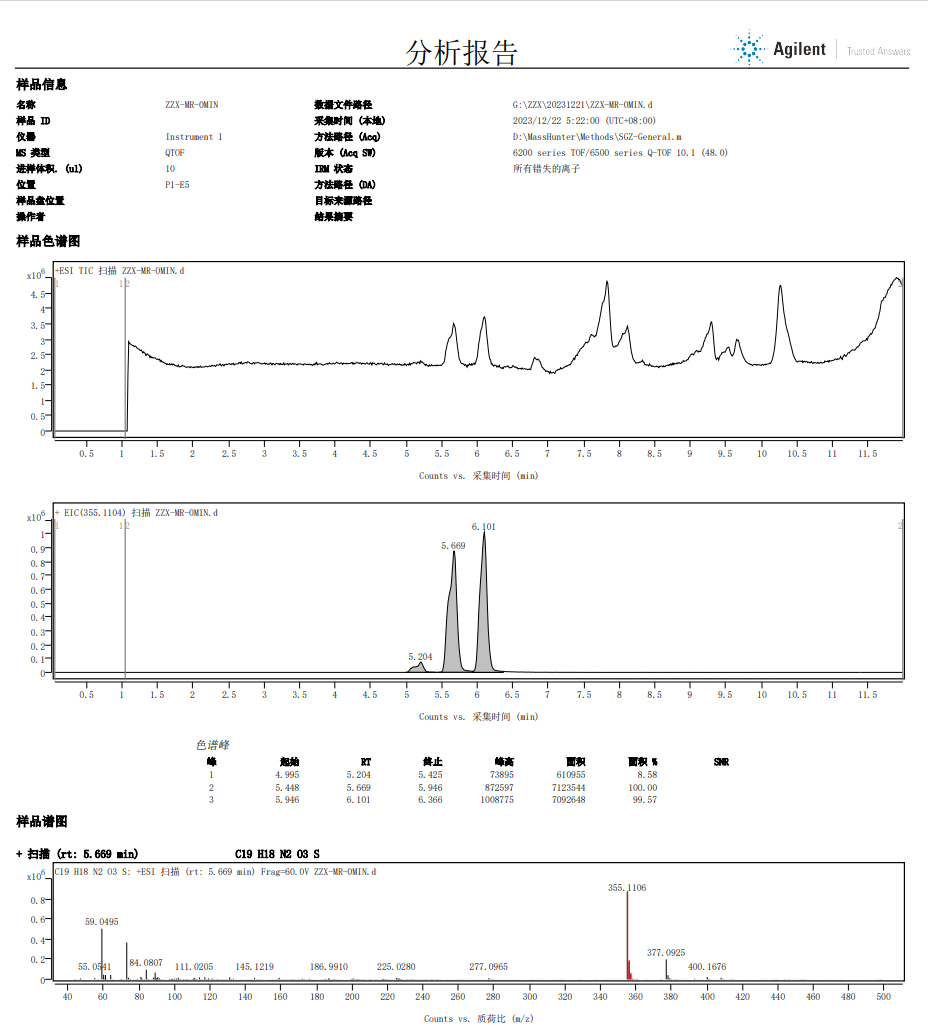


**HR-ESI-MS spectra of Mei-BME**
